# Conformational Snapshots of CydDC in a Native Lipid Bilayer Coupling Heme Transport to Antibiotic Resistance

**DOI:** 10.1002/advs.76081

**Published:** 2026-06-11

**Authors:** Lili Yang, Changbin Zhang, Mengyuan Lyu, Yongbo Luo, Jierou Zhang, Yujiao Chen, Lintao Luo, Wen Qiao, Xiangyangpeng Li, Yu Zhou, Zhongling Wei, Yuling Xiao, Qian Niu, Juan Zhou, Guanglin He, Binwu Ying, Zhaoming Su, Hao Chen, Xiaodi Tang, Haohao Dong

**Affiliations:** ^1^ Department of Laboratory Medicine State Key Laboratory of Biotherapy and Cancer Center National Clinical Research Center for Geriatrics West China Hospital Sichuan University Chengdu China; ^2^ Department of Forensic Medicine College of Basic Medicine Chongqing Medical University Chongqing China; ^3^ Center For Archaeological Science Sichuan University Chengdu China

**Keywords:** CydDC, drug resistance, evolutionary, heme

## Abstract

Heme is an essential cofactor in numerous biological processes, including bacterial respiration. The ABC transporter CydDC facilitates the assembly and maturation of the cytochrome *bd* terminal oxidase by exporting heme and has been implicated in antibiotic resistance in bacteria. However, the dynamic conformations of CydDC in a native‐like lipid bilayer remain unresolved, and its resistance mechanism is still elusive. Here, we determined high‐resolution cryo‐electron microscopy structures of nanodisc‐reconstituted CydDC in the apo, nucleotide‐bound and heme‐bound states, providing direct structural evidence for its substrate‐stimulated hydrolysis mechanism. In vivo and in vitro biochemical characterization identified key residues of CydDC that are critical for substrate binding and transport. Bioinformatics analysis further demonstrated that CydDC is highly conserved across bacterial species. Transcriptomic profiling of *cydC/D* in antibiotic‐resistant strains showed that elevated expression of *cydC/D* correlates with increased antibiotic resistance. Moreover, mutations at the heme‐binding sites altered bacterial susceptibility to multiple antibiotics, suggesting that the exporting activity of CydDC may also contribute directly to drug resistance. Together, these findings provide mechanistic insights into CydDC‐mediated heme transport and potential drug efflux, and inform the development of antimicrobial strategies targeting the respiratory chain.

## Graphical abstract

Phylogenetic and biochemical analyses of the heme transporter CydDC reveal its functional conservation throughout bacterial evolution and demonstrate its unique asymmetric allosteric mechanism. Furthermore, impairment of CydDC function directly affects bacterial antibiotic resistance and likely compromises antibiotic efficacy through drug efflux. This study highlights CydDC as a key component in bacterial metabolic processes and provides new perspectives for developing antimicrobial agents.

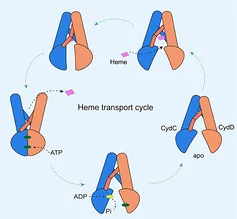

## 1 Introduction

The escalating global challenge of antimicrobial resistance (AMR) poses a severe threat to public health, underscoring the urgent need for novel antibiotics [1]. A comprehensive understanding of bacterial life processes is crucial for developing new antibiotics and addressing the growing burden of infectious diseases. Bacterial respiration provides the energy necessary for both survival and pathogenicity [2], with cytochrome *bd* acting as a terminal oxidase in the respiratory chain [3], which is a key factor in maintaining bacterial viability under stress [4, 5, 6, 7]. Cytochrome *bd* is a respiratory chain component exclusive to bacteria and lacks homologs in mitochondria [8]. Both the complex itself and key proteins involved in its assembly have emerged as strategic targets for disrupting bacterial energy metabolism [9, 10]. However, current cytochrome *bd* inhibitors are limited to quinone‐like compounds such as Aurachin D [11], which pose toxicity risks due to their structural resemblance to substrates in mammalian mitochondria. Consequently, alongside ongoing efforts to optimize existing inhibitors, exploring novel targets associated with cytochrome *bd* represents a promising therapeutic strategy against drug‐resistant bacteria.

Research indicates that the ABC transporter CydDC is essential for the maturation and assembly of cytochrome *bd* [12]. Furthermore, loss of CydDC function affects the operation of other respiratory chain complexes, making it a critical role in energy metabolism [13]. Increasing evidence shows that CydDC has a widespread impact on various bacterial physiological processes, including thiol metabolism, nitric oxide tolerance, pH regulation, and even immune evasion [14, 15, 16, 17]. Most importantly, it plays a crucial role in maintaining bacterial redox balance. Studies have proposed that CydDC functions as a glutathione transporter with cystine reductase activity, rather than the initially suggested cysteine transporter [18, 19, 20]. A long‐standing hypothesis proposed that CydDC might transport heme to facilitate the maturation of cytochrome *bd* [14], yet direct evidence remained elusive for years. Only recently have the structural studies of CydDC in complex with the heme provided direct evidence for this function [21, 22]. Additionally, emerging evidence indicates that the inactivation of CydD increases bacterial resistance to aminoglycosides, chloramphenicol, and rifampicin [20, 23], but enhances bacterial susceptibility to certain β‐lactams and quinolones [24]. Studies on the potential involvement of CydDC in the drug resistance of *Mycobacterium tuberculosis* (*M. tb*) have broadened the scope of its impact [25], suggesting functional conservation across different bacterial species. Meanwhile, CydDC has also been implicated in antimicrobial vaccines research [16]. These findings indicate that CydDC connects small molecule transport, energy metabolism, and oxidative stress response, serving as a pivotal component in deciphering the complex metabolic network of bacteria.

Currently studies have elucidated multiple CydDC conformational states from various species under detergent conditions, establishing key features of heme transport [21, 22]. Despite these advances, several critical aspects remain insufficiently understood. The relationship between CydDC‐mediated heme transport and bacterial antibiotic resistance remains poorly defined. It is unclear whether the complete dynamic conformational of CydDC can be recapitulated under native lipid nanodisc conditions, and the evolutionary relationships of CydDC across species have not been systematically characterized. To address these questions, we first employed bioinformatic analyses to explore the evolutionary relationships of CydDC among diverse bacterial species, broadening our understanding of its biological functions. Furthermore, to elucidate the molecular mechanism of CydDC, we determined the high‐resolution cryo‐electron microscopy (cryo‐EM) structure of CydDC using lipid nanodiscs that closely mimic the native membrane environment, accurately capturing its conformational dynamics during heme translocation and identifying key heme‐binding residues. Subsequently, we conducted in vivo and in vitro experiments to examine the relationship between these binding sites and bacterial antibiotic resistance, providing critical support for future targeted drug design.

## 2 Results

### 2.1 *CydC/D* Exhibits Evolutionary Conservation across Species

**FIGURE 1:**
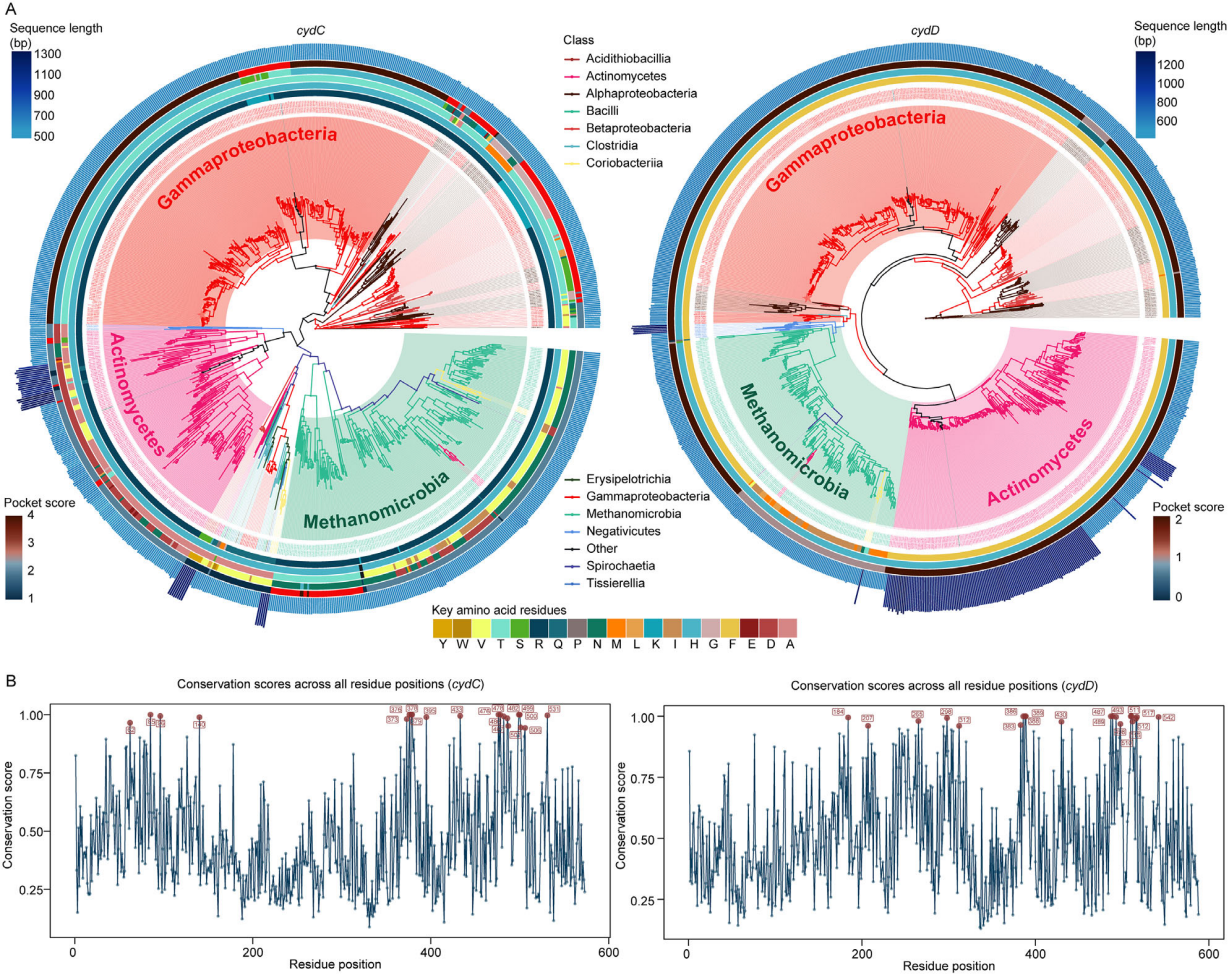
Maximum‐likelihood phylogenetic tree and conservation analysis of *cydC* and *cydD* orthologs across representative prokaryotic lineages. A) Branches were colored according to major prokaryotic groups. Concentric rings (from inner to outer) display the residue types of interest, pocket scores, and sequence lengths. The left panel highlights residues R81, H85, T88, and H132 of cydC, and the right panel shows residues F247 and H312 of cydD. *Caulobacter vibrioides* was used as the outgroup. B) Sequence conservation scores were calculated based on per‐residue information entropy and normalized to a scale of 0–1, where higher values indicate higher conservation. The conservation profiles are plotted along the amino acid positions of the *E. coli* reference sequence, with highly conserved residues labeled. Top 20 conserved sites were highlighted in red.

To elucidate the evolutionary basis of cydC/D‐mediated substrate transport and its functional conservation across bacteria, we conducted a comprehensive phylogenetic and sequence conservation analysis of *cydC* and *cydD* orthologs from 913 representative prokaryotic species (Table S1). Both genes exhibited highly constrained coding lengths across taxa, with median lengths of 574 bp for *cydC* (*n* = 906) and 588 bp for *cydD* (*n* = 997), and coefficients of variation of 0.199 and 0.325, respectively, indicating strong structural conservation (Figure S1). A maximum‐likelihood phylogenetic reconstruction revealed that *cydC* and *cydD* share congruent evolutionary trajectories (Figure 1A). At the order level, both genes first diverged within *Gammaproteobacteria*, followed by *Actinomycetes* and *Methanomicrobia*, consistent with their functional coupling as a heterodimeric transporter complex. We further quantified pocket scores for all residues of both proteins across species. The average completeness scores were 0.955 ± 0.038 for *cydC* and 0.944 ± 0.042 for *cydD*, reflecting a high degree of structural integrity and limited domain loss (Table S2). Such pervasive conservation implies that the functional core of the cydD/C complex has been maintained under strong purifying selection to preserve the essential framework required for substrate recognition and binding. Based on the conservation profiles, several residues showing consistently high conservation across taxa were identified and subsequently selected as candidate functional sites for downstream structural and biochemical validation (Figure 1B). These results emphasize the broad relevance and potential of CydDC as a drug target.

### 2.2 Precise Dynamic Reconstruction of CydDC in Lipid Nanodiscs

**FIGURE 2:**
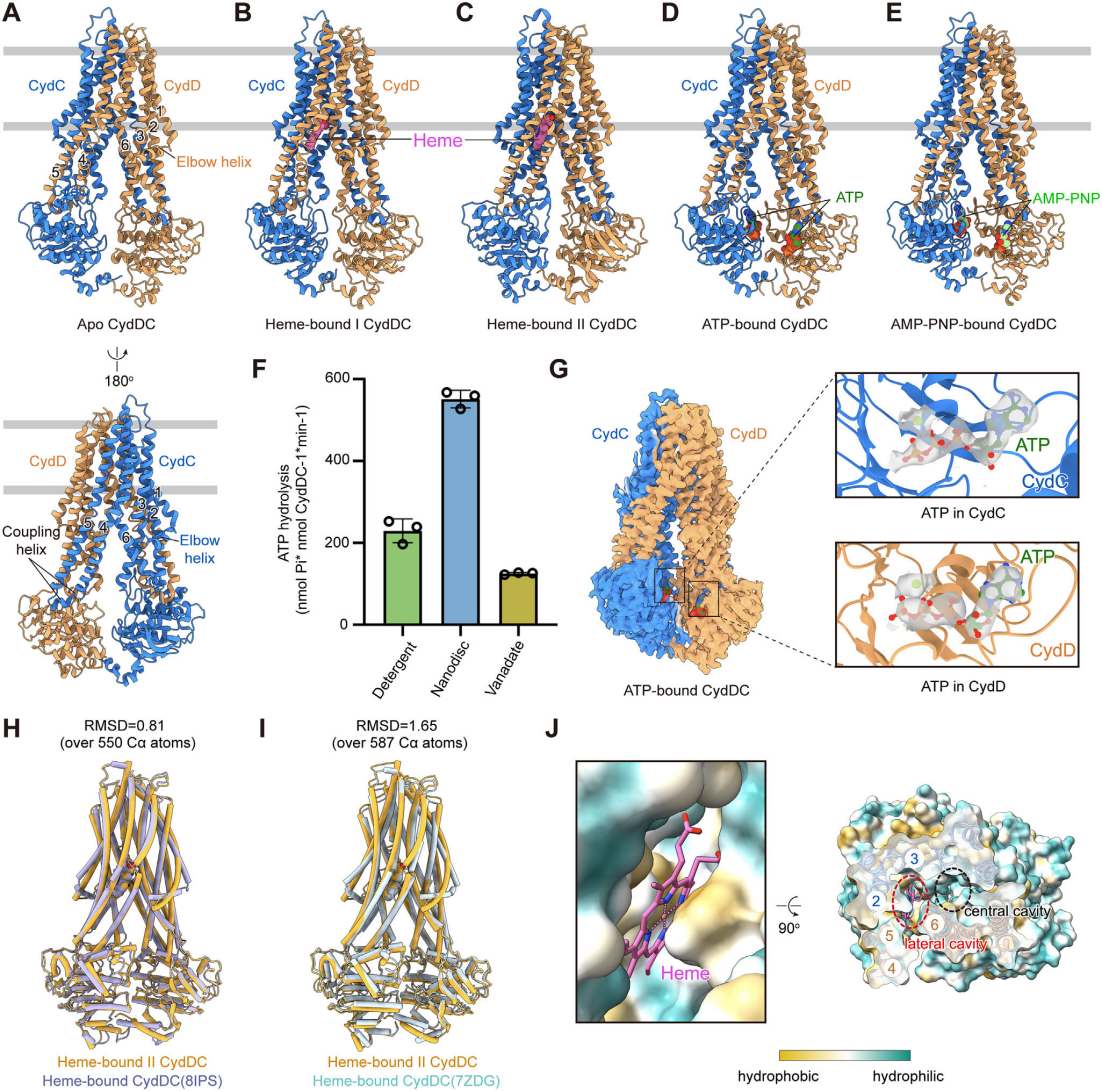
Cryo‐EM structures of CydDC in nanodisc with different substrates. A–E) Cartoon model of CydDC in apo (A), heme‐bound (B, C), ATP‐bound (D) and AMP‐PNP‐bound (E) states. CydC, CydD, heme, ATP and AMP‐PNP are colored with blue, sandy brown, pink, sea green and light green, respectively. F) ATPase activity of CydDC under different conditions. G) Map of ATP‐bound CydDC. H, I) Superimposition of heme‐bound CydDC (this study) with reported structures of CydDC. J) Hydrophobic surface of heme‐bound I CydDC.

To faithfully recapitulate the dynamic substrate‐transport cycle and capture transient intermediate conformations, we overexpressed and purified the CydDC complex from *E. coli*, reconstituted it into lipid nanodiscs, and incubated it with ATP or AMP‐PNP, MgCl_2,_ and heme (Figure S2). We successfully captured the cryo‐EM structures of CydDC in multiple states: apo (3.71 Å), ATP‐bound (2.82 Å), AMP‐PNP‐bound (2.93 Å), heme‐bound I (3.40 Å), and heme‐bound II (3.41 Å) (Figure 2A–E; Figures S3 and S4). ATPase activity assays showed that the activity of CydDC in lipid nanodiscs was 2.4 times higher than in detergent (Figure 2F), indicating that lipid nanodiscs preserve CydDC activity in vitro.

Consistent with previous findings [21, 22], CydDC is a heterodimer composed of CydC and CydD subunits, each containing conserved nucleotide‐binding domains (NBDs) and transmembrane domains (TMDs). The TMD of each subunit consists of six α‐helices, with an elbow helix (EH) at the N‐terminus of TM1, aligned parallel to the membrane plane (Figure 2A–E). TM4 and TM5 intertwine with TM1‐3 and TM6 of the other subunit (Figure 2A–E), exhibiting the typical structural features of the type IV ABC transporter. Despite capturing multiple ligand‐bound states, all structures displayed an inward‐facing (IF) conformation of the TMDs. Notably, Zhu et al. reported an outward‐facing (OF) conformation of CydDC from *Mycobacterium smegmatis* [22], which may reflect species‐specific differences in the stability or conformational preferences of CydDC.

Our apo and AMP‐PNP‐bound CydDC structures are highly consistent with the previously reported structures (PDB: 7ZD5, 7ZDK) (Figure S5A,B) [21]. In addition, we determined the ATP‐bound structure of wild‐type CydDC, which has not been reported in prior detergent‐based studies (Figure 2G). Upon incubation with heme alone, we captured two distinct states of heme‐bound CydDC (heme‐bound I and heme‐bound II) (Figure 2B,C). Under comparable ligand conditions in detergent, Wu et al. described one heme‑bound conformation (PDB: 7ZDG) and observed further conformational heterogeneity only after nucleotide addition [21]. Structural comparison shows that heme‐bound II resembles the structures PDB: 8IPS and 7ZDG (Figure 2H,I) [21, 22], whereas heme‐bound I represents a previously uncharacterized nucleotide‐free heme‐bound conformation, expanding the known conformational landscape of CydDC. In all heme‐bound structures, a heme molecule consistently binds in the lateral cavity formed by TM2‐3 of CydC and TM4‐6 of CydD, rather than in the central cavity (Figure 2J). This binding mode is similar to that observed in the iron (III) importer YbtPQ (PDB: 6P6J) [26] (Figure S5C). We propose that this unique localization may be attributed to the hydrophobic nature of heme, as the lateral cavity exhibits significantly stronger hydrophobicity than the central cavity (Figure 2J). The central cavity might only serve as a transient channel during heme transport.

### 2.3 Heme Binding Induces Conformational Changes in CydDC

**FIGURE 3:**
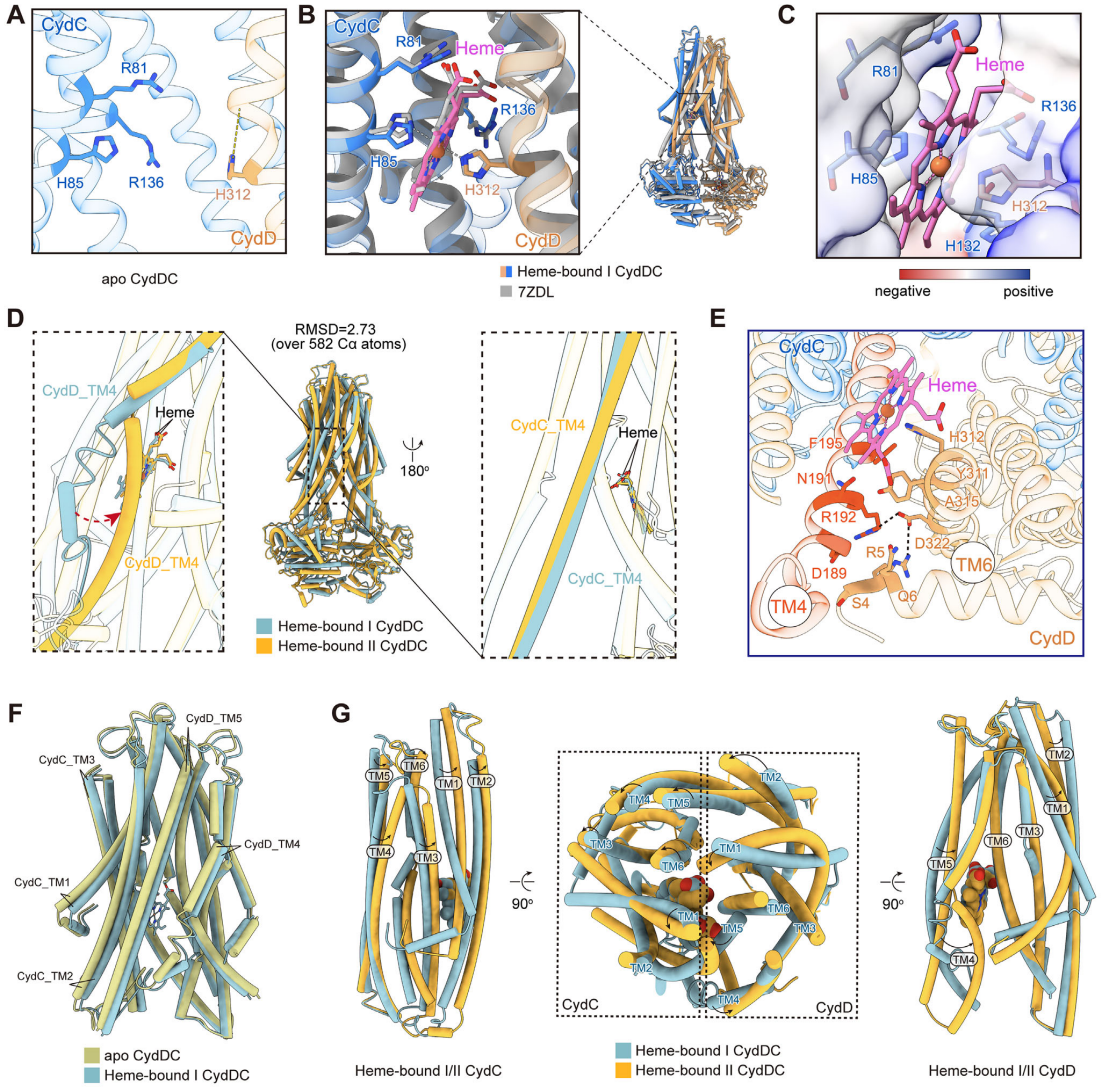
Heme binding induces conformational changes in CydDC. A, B) Interactions between CydDC and heme. Hydrogen bonds are shown as yellow dashed lines. C) Electrostatic surface of heme‐bound CydDC. D) Superimposition of heme‐bound I and heme‐bound II CydDC. E) Interactions between TM4, TM6 and elbow helix of CydD. TM4 of CydD is highlighted with red orange. Salt bridges are shown as black dashed lines. F, G) Rotation conformational changes of TMDs between apo and heme‐bound I (F), heme‐bound I and heme‐bound II CydDC (G).

In heme‐bound structures, multiple surrounding residues form extensive interactions to stabilize heme accommodation (Figure S6A,B). Notably, during the transition from apo state to heme‐bound I, the side chain of CydD^H312^ reorients toward the lateral cavity and, together with CydC^H85^, provides axial coordination to the iron center of heme, consistent with previous structural observations (Figure 3A) [21]. In contrast, the CydD^H276^ of *Mycobacterium smegmatis* CydDC (corresponding to *E. coli* CydD^H312^) consistently points toward the heme‐binding cavity, indicating differences in heme recruitment among different bacterial species [22]. In addition, the CydC^R81^ forms an electrostatic interaction with the propionate group upon heme binding (Figure S6A,B). In heme‑bound I, the side chain of CydD^R136^ shifts upward relative to the apo conformation and interacts with the other propionate group (Figure 3A,B), an interaction absent in the previously reported structure PDB: 7ZDL (Figure 3B) [21]. R81 and H85 of CydC primarily function in initial heme recruitment, while R136 of CydC and H312 of CydD further stabilize bound heme. Together, with H132 of CydC, these positively charged residues establish a favorable electrostatic environment to secure heme binding within the lateral cavity (Figure 3C). The heme‑bound I structure provides additional conformational details to the dynamic process of heme binding to CydDC.

Superimposition of heme‐bound I and heme‐bound II reveals a RMSD of 2.73 over 582 Cα atoms (Figure 3D). A dramatic conformational difference in TM4 of CydD was observed, which governs the gating of the lateral gate (Figure 3D). The gated channel is exclusively present in CydD but not in CydC (Figure 3D). In heme‐bound I, the lateral gate remains open, with the middle segment of CydD^4^ protruding outward, forming a passage wide enough for heme entry (Figure 3D). In heme‐bound II, CydD^4^ moves significantly and forms a complex interaction network with heme, TM6, and the EH of CydD, locking the lateral gate into a stable closed conformation (Figure 3E). Our series of structurally resolved intermediates under identical conditions provides direct structural snapshots of substrate‐triggered sequential conformational transitions. Structural analysis of type IV heterodimeric ABC transporters, including TmrAB, BmrCD, YbtPQ, IrtAB, and TM287/288, reveals that TM4 or TM6 consistently plays a critical role in substrate channel gating (Figure S7A–E) [26, 27, 28, 29, 30]. This recurring feature likely represents a common mechanism among this class of ABC transporters.

Compared with the apo structure, heme binding in heme‐bound I induces only minor conformational changes, reflected by a slight counterclockwise shift in TM1‐3 of CydC and TM4‐5 of CydD toward the lateral cavity (Figure 3F; Figure S7F). In contrast, heme binding in the heme‐bound II state induces pronounced rearrangements in these helices (Figure 3G), indicating a stepwise structural transition from apo to heme‐bound I, and ultimately to the heme‐bound II upon heme engagement. This progressive structural remodeling lays a conformational foundation for subsequent outward‐facing exit opening and substrate release. The overall TMD rearrangement trend is consistent with the findings of apo to heme‐coordinated to heme‐confined reported by Wu et al. (Figure S7G,H) [21]. CydDC shows conserved conformational behavior observed in both detergent and nanodisc environments cross‐validates the physiological relevance of the proposed transport mechanism.

### 2.4 Substrate‐Dependent ATP Hydrolysis Mechanism

**FIGURE 4:**
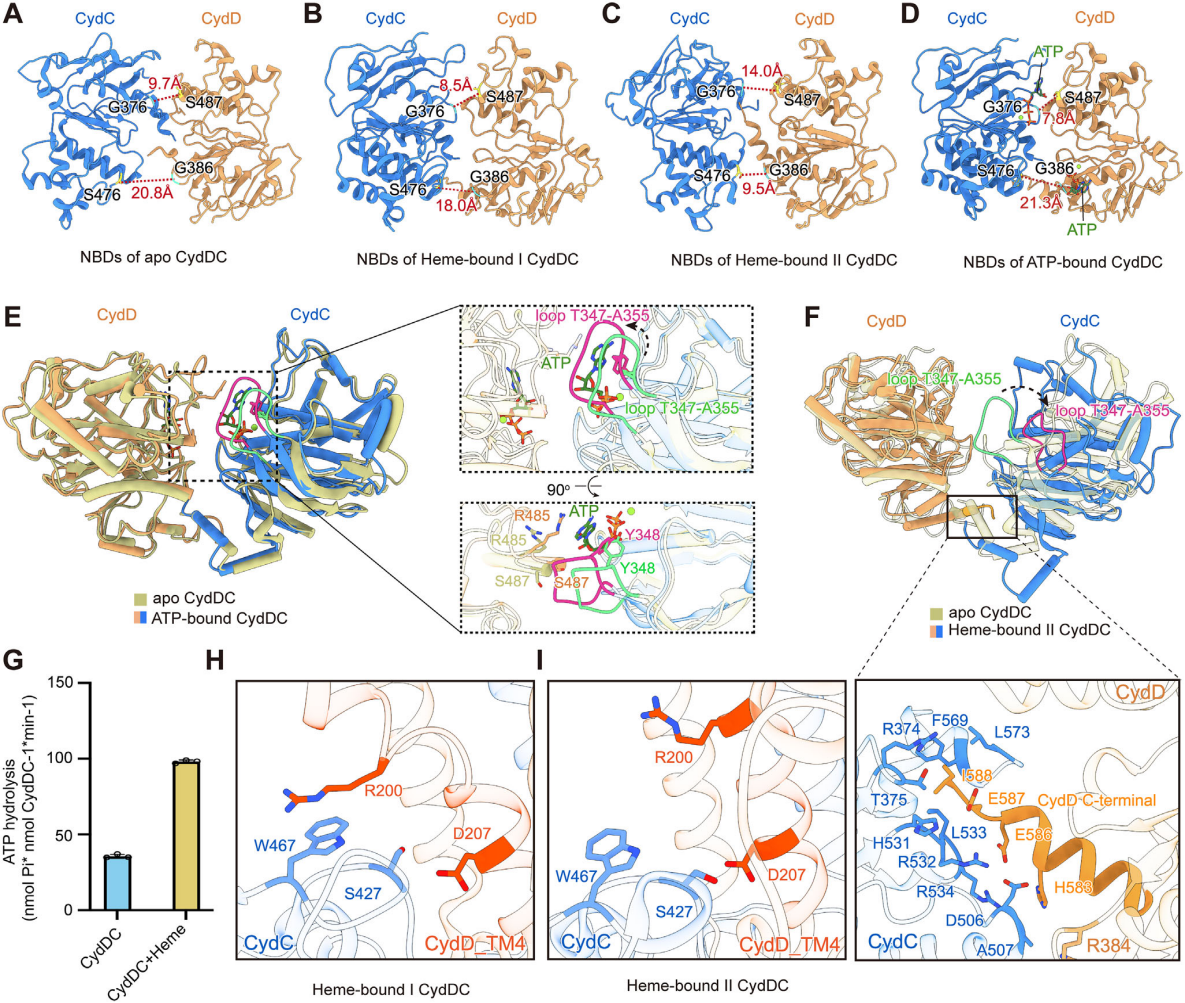
Interactions of NBDs in CydDC. A–D) Distances between the NBDs of CydDC in apo (A), heme‐bound I (B) and heme‐bound II (C), ATP‐bound (D). Conserved glycine and serine are highlighted with cyan and yellow. E) Superimposition of apo and ATP‐bound CydDC. F) Superimposition of apo and heme‐bound II CydDC. The C‐terminal of CydD is highlighted with orange. G) ATPase activity of CydDC and CydDC with heme. H, I) Interactions between NBDs and TM4 of CydD in heme‐bound CydDC.

In the apo state, the NBDs exhibit an asymmetric arrangement, reflected by distinct distances between the conserved glycine residue in Walker A (G373‐S380 of CydC; G383‐S390 of CydD) and the serine residue in the signature motif (L475‐E479 of CydC; L486‐Q490 of CydD) (Figure 4A). This asymmetric arrangement has not been reported for other ABC transporters of the same class (Figure S8A–E). The NBDs progressively adopt a more symmetric arrangement from apo to heme‑bound I and further to heme‑bound II, reflecting the mechanical coupling between the TMDs and NBDs (Figures 3F,G and 4A–C). Specially, ATP or AMP‐PNP binding does not induce canonical dimerization of NBDs, which remain asymmetric in our experimental condition (Figure 4D; Figure S8F). In the ATP‐bound state, loop T347‐A355 of CydC approaches the NBD of CydD compared with apo state (Figure 4E), and enwraps the bound ATP molecule, a feature that can be observed in all structures that nucleotide binding to CydC but not to CydD alone [21]. The side chain of CydD^R485^ simultaneously moves closer and, together with the opposing CydC^Y348^, clamps the adenosine (Figure 4E). A similar structural rearrangement is observed in both our determined AMP‑PNP‑bound structure and previously reported counterparts [21].

In heme‑bound II, the NBDs adopt a more symmetrical arrangement with relatively evenly spaced interfaces (Figure 4C). The C‐terminal region of CydD extends into the NBD space of CydC, forming tight contacts that appear to “pull” the NBD of CydC closer (Figure 4F; Figure S8G–I). Concurrently, the loop T347‐A355 of CydC retracts (Figure 4F), potentially providing a pathway for ATP binding or ADP release [21]. Although CydD contains a degenerate nucleotide‐binding site and only the ATP binding to CydC is hydrolyzed, the symmetry remodeling of NBDs is required for productive dimerization and efficient ATP turnover. Heme binding therefore primes the NBDs to adopt a catalytically competent configuration upon nucleotide engagement. Biochemical assays confirm that heme stimulates the ATPase activity of CydDC and enhances hydrolysis efficiency [14, 21], consistent with our ATPase activity assay results (Figure 4G). Our ATP‑bound and heme‑bound II structures provide direct structural evidence for the substrate‑dependent ATP hydrolysis mechanism of CydDC. A similar substrate‐dependent NBD‐priming mechanism has been reported for the type VII ABC transporter LolCDE [31].

We identified interactions between residues R200 and D207 on TM4 of CydD and W467 and S427 on the NBD of CydC in the apo, heme‐bound I and ATP/AMP‐PNP‐bound structures (Figure 4H; Figure S8J–L). In the heme‐bound II structure, however, the side chain of CydD^R200^ shifts upward, disrupting its contact with CydC^W467^ (Figure 4I). This disruption releases the CydD^4^ constraint imposed by interactions with NBD of CydC, allowing CydD^4^ to undergo a substantial displacement upon heme binding. Together with the aforementioned TMDs changes, this shift brings the signature motif of CydC closer to the Walker A motif of CydD, while the Walker A of CydC moves away from the signature motif of CydD (Figures 3F,G and 4A–D), facilitating ATP binding and NBDs dimerization.

### 2.5 Heme Transport Disruption Affects Bacterial Antibiotic Resistance

**FIGURE 5:**
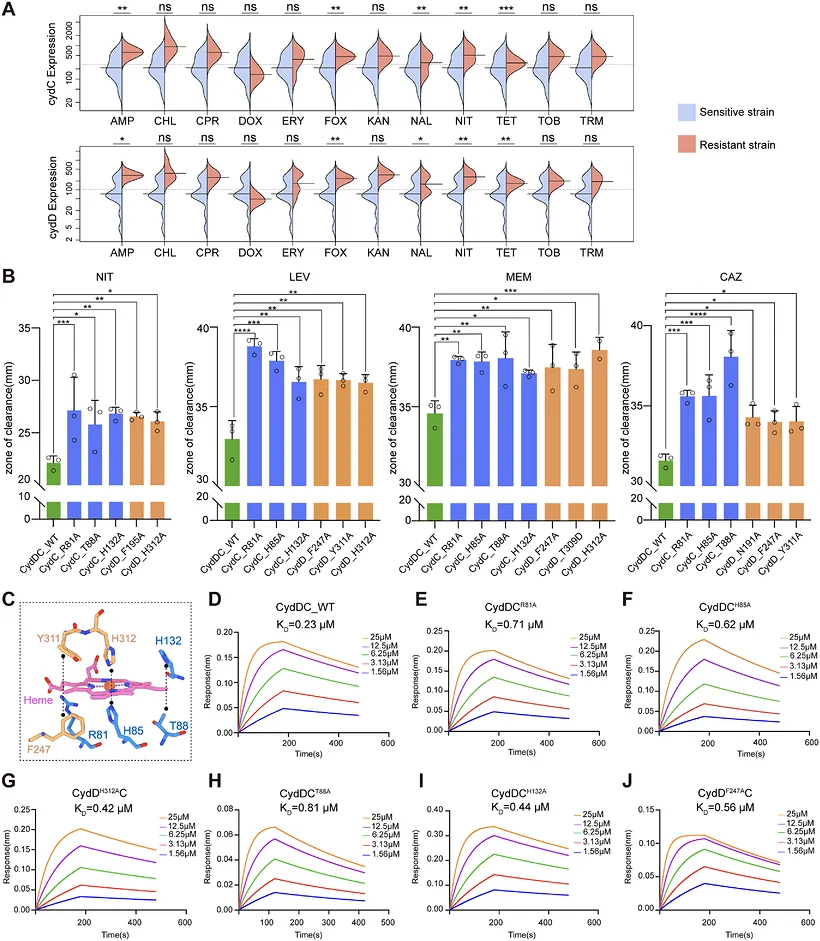
Expression analysis of CydDC and susceptibility testing. A) Expression levels of *cydC/D* in different resistant bacteria and sensitive bacteria. AMP: ampicillin; CHL: chloramphenicol; CPR: cefprozil; DOX: doxycycline; ERY: erythromycin; FOX: cefoxitin; KAN: kanamycin; NAL: nalidixic acid; NIT: nitrofurantoin; TET: tetracycline; TOB: tobramycin; TRM: trimethoprim. Adjusted *p* values (*p*_adj_) are marked with black asterisks for significant differences. ns: no significant differences. *p_adj_ < 0.05, **p_adj_ < 0.01, ***p_adj_ < 0.001, ****p_adj_ < 0.0001. B) Zone of inhibition about CydDC mutant with different antibiotics. LEV: levofloxacin; MEM: meropenem; CAZ: ceftazidime. ns: no significant differences. WT: wildtype. WT CydDC is colored with green; Mutants with mutations in CydC and CydD are colored with blue and orange, respectively. *p_adj_ < 0.05, **p_adj_ < 0.01, ***p_adj_ < 0.001, ****p_adj_ < 0.0001. C) Key residues interact with heme. D–J) Affinity test results for wildtype CydDC and mutants with heme by biolayer interferometry.

Recent studies have highlighted the role of CydDC in bacterial resistance to multiple antibiotics [20, 23, 24]. CydDC shares mechanistic similarities with other ABC transporters linked to multidrug resistance, such as TmrAB, BmrCD, and TM287/288, which prompted our investigation into its involvement in multidrug resistance. Transcriptomic profiling of drug‐resistant *E. coli* strains uncovered that both *cydC* and *cydD* were consistently and significantly upregulated in response to ampicillin (AMP), cefoxitin (FOX), nalidixic acid (NAL), nitrofurantoin (NIT), and tetracycline (TET) (Figure 5A), indicating their functional synergy and potential roles in resistance to these distinct drug classes. To determine whether this phenotype is associated with CydDC‐mediated heme transport, we performed antibiotic susceptibility assays of CydDC heme‐binding site mutants. The antibiotics tested included those that showed significant differences in prior results, alongside several commonly used in clinical practice. These mutations didn't affect protein expression or complex formation (Figure S9A). Specifically, no significant differences in inhibition zones were observed between mutant and wild‐type (WT) strains when treated with TET or NAL (Figure S9B,C), In contrast, mutants CydDC^R81A^, CydDC^T88A^, CydDC^H132A^, CydD^F195A^C, and CydD^H312A^C showed significantly larger inhibition zones on NIT plates, indicating increased drug susceptibility (Figure 5B; Figure S9D). Similar increased susceptibility to levofloxacin (LEV) was observed in mutants CydDC^R81A^, CydDC^H85A^, CydDC^H132A^, CydD^F247A^C, CydD^Y311A^C, and CydD^H312A^C (Figure 5B; Figure S9E). Mutants CydDC^R81A^, CydDC^H85A^, CydDC^T88A^, CydDC^H132A^, CydD^F247A^C, CydD^T309D^C, and CydD^H312A^C also displayed increased susceptibility to meropenem (MEM) (Figure 5B; Figure S9F), while strains containing mutants CydDC^R81A^, CydDC^H85A^, CydDC^T88A^, CydD^N191A^C, CydD^F247A^C, and CydD^Y311A^C showed increased susceptibility to ceftazidime (CAZ) (Figure 5B; Figure S9G).

Through these antimicrobial susceptibility assays, we identified six residues, R81, H85, T88 and H132 of CydC, F247 and H312 of CydD, that broadly contribute to bacterial resistance against these antibiotics (Figure 5B,C). We further evaluated the impact of these residues on heme binding. The *K*_D_ values for mutants CydDC^R81A^, CydDC^H85A^, and CydD^H312A^C with heme were 0.71, 0.62, and 0.42 µm, respectively, compared to 0.23 µm for wild‐type (WT) CydDC (Figure 5D–G), indicating their critical roles in heme binding. These residues, which are positively charged, form multiple hydrogen bonds with heme (Figure S10A), suggesting that the electrostatic environment plays a significant role in heme binding. The interaction of CydC^T88^ with heme was only observed in the heme‐bound I state, where it cooperates with CydC^H132^ to interact with the same pyrrole ring of heme. The *K*_D_ values for mutants CydDC^T88A^ and CydDC^H132A^ were 0.81 and 0.44 µm, respectively (Figure 5H,I). These results indicated that CydC^R81^, CydC^H85^and CydC^T88^, stabilizing heme binding during the initial stages, exert a stronger influence on the physiological function of CydDC compared to CydC^R136^, CydD^H312^ and CydC^H132^, which are in agreement with structural data. Interestingly, CydC^T88^ and CydC^H132^ are positioned opposite each other, mirroring the symmetric arrangement of the axial residues CydC^H85^ and CydD^H312^. A similar symmetric distribution is observed between CydD^F247^ and CydD^Y311^, which form π‐π stacking interactions with a heme pyrrole ring (Figure 5C). This near‐symmetric spatial arrangement is crucial for stable heme binding. Among these, CydD^F247^ forms a T‐shaped π–π stacking interaction with the pyrrole ring, exhibiting stronger binding forces compared to CydD^Y311^. Consistent with this, the mutant CydD^F247A^C showed altered resistance to a broader range of antibiotics (Figure 5B). Compared to WT CydDC, the mutant CydD^F247A^C showed reduced heme affinity, with a *K*_D_ value of 0.56 µm (Figure 5J). These residues are distributed around the lateral cavity formed by CydC^2^, CydC^3^, CydD^5^, and CydD^6^, creating a “molecular scaffold” that maintains the spatial orientation of heme. Importantly, residues R81 and H85 of CydC, F247 and H312 of CydD are highly conserved across diverse pathogens, including Gram‐positive species (Figure 1), underscoring the conserved mechanism of CydDC and supporting its potential as a target for broad‐spectrum therapeutic development.

The mutants exhibited *K*_D_ values of 0.42–0.81 µm, representing a 1.8‐ to 3.5‐fold reduction in affinity (Figure 5E–J). To correlate in vitro affinity changes with physiological function, we calculated the heme‐binding occupancy at a physiologically relevant concentration of 1 µm, a standard concentration applied in bacterial heme uptake assays [32]. The fractional occupancy decreases from ≈81% for wild‐type to 55–70% for the mutants regarding to the equation). In fact, the physiological concentration of heme within bacterial cells may be even lower due to its toxicity [33], and the functional impact of the mutations on CydDC is likely to be further amplified in vivo. This substantial reduction in binding efficiency may directly contribute to severe impairment of bacterial physiological processes.

## 3 Discussion

**FIGURE 6:**
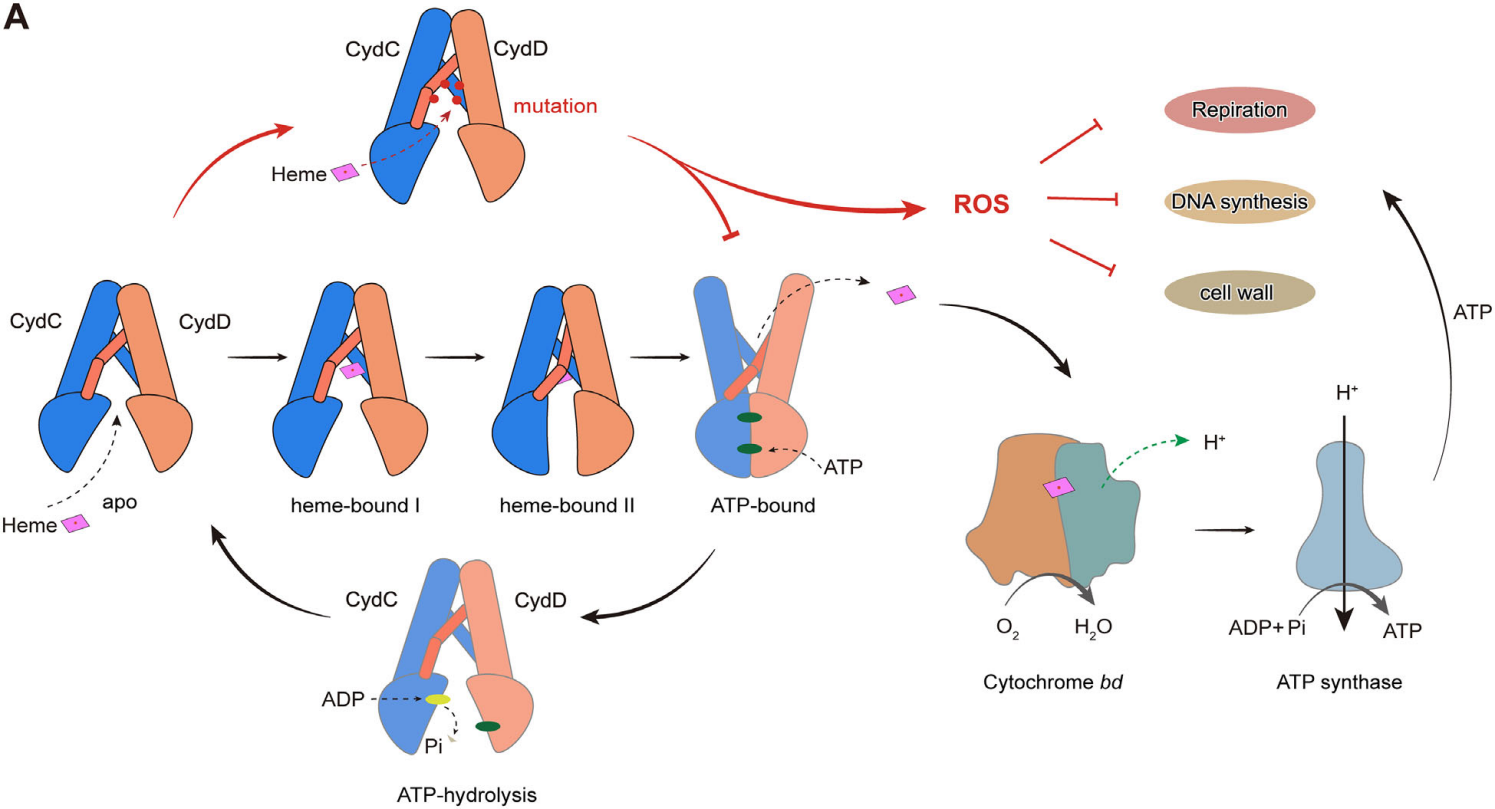
Schematic diagram of the mechanism of heme transported by CydDC. In the apo state, the lateral gate of CydDC remains open, awaiting heme entry. Heme binding induces shifts in the TMDs due to intermolecular forces and distances reducing of NBDs. Subsequent ATP binding triggers NBD dimerization, rotation of the TMDs, opening of the periplasmic exit, and heme extrusion. CydDC then gradually returns to the apo state, ready for the next cycle. The normal transport of heme ensures the progression of respiratory reactions, providing energy for bacterial life activities. Impaired function of the CydDC in heme transport leads to oxidative stress responses within bacterial cells, thereby affecting various downstream physiological processes. The structures determined in this study are outlined with black solid lines in the heme transport cycle of CydDC.

As a key component of the bacterial respiratory chain, CydDC plays a crucial role in various physiological processes, including resistance to multiple antibiotics (Figures 5A,B and 6). To further elucidate its essential function in bacterial life, we first constructed an evolutionary profile of CydDC using bioinformatics. Our analysis revealed that the structural and functional framework of CydDC is highly conserved across bacterial evolution, with no significant distinction between Gram‐negative and Gram‐positive species. This widespread conservation underscores the profound physiological relevance of CydDC and supports its potential as a promising target for broad‐spectrum antibiotic development.

To guide the precise design of antimicrobial agents, we employed lipid nanodiscs to mimic the native biological membrane environment, enabling accurate reconstruction of the dynamic heme‐transport cycle by CydDC. Integrating our findings with those of Wu et al. and Zhu et al. [21, 22], we gained new insights into the molecular mechanism of heme transport by CydDC. In the apo state, the lateral gate of CydDC remains open, awaiting heme entry. Initial heme binding (heme‐bound I) induces minor shifts in the TMDs due to intermolecular forces. This shift disrupts the critical interaction between CydD^4^ and the NBD (CydD^R200^‐CydC^W467^), leading to a dramatic conformational change in CydD^4^ that closes the lateral gate (heme‐bound II). This closed conformation is further stabilized by intermolecular networks involving the elbow helix and TM6 of CydD. Accompanying gate closure, the two NBDs transition from an asymmetric to a more symmetric arrangement, priming the CydC nucleotide‐binding site for ATP engagement and facilitating subsequent NBD dimerization. Notably, our heme‐bound I structure defines distinct functional roles for key residues: R81 and H85 of CydC are primarily responsible for initial heme recruitment, whereas R136 of CydC and H312 of CydD function to stabilize bound heme. Subsequent ATP binding promotes NBD dimerization, TMD rotation, opening of the periplasmic exit gate, and eventual heme release (Figure 6).

This asymmetric change in the NBDs of CydDC is not observed in other ABC transporters with so‐called degenerate nucleotide‐binding sites, such as TmrAB, BmrCD, and TM287/288, highlighting the uniqueness and mechanistic complexity of CydDC. As heme transporters, the CcmABCD complex operates through intricate coordination among its components [34], and CcsBA exhibits bifunctional characteristics [35]. The outward‐facing (OF) conformation of *M. tb* CydDC reported previously was also obtained from a dimeric state [22]. We proposed that the difficulty in stabilizing and capturing the OF conformation of CydDC in vitro might be due to the absence of auxiliary downstream proteins. Moreover, uncoupling between ATP binding and complete NBD closure has been observed in multiple independent systems. In PCAT1, Zhang et al. found that the fully closed NBD conformation is not a stable state under active turnover conditions [36]. The classical importer BtuCD‑F requires disulfide cross‑linking to “lock” its ATP‑bound state [37], which itself suggests the inherent dynamics or instability of this conformation under native conditions. Together, these observations point toward a more complex energy model, enriching our understanding of the functional diversity of ABC transporters.

Although research on the role of CydDC in bacterial antibiotic resistance is emerging, the underlying mechanisms remain unclear and require further exploration. In this study, we identified six key residues crucial for heme transport. Mutations of these residues are likely to alter the local hydrophobic and electrostatic microenvironment, destabilizing the hydrophobic core of the heme‐binding pocket and reducing heme affinity for CydDC and heme. These mutations broadly influence resistance to NIT, MEM, LEV, and CAZ, which belong to the β‐lactam (NIT, MEM), quinolone (LEV), and cephalosporin (CAZ) classes. It is well‐established that β‐lactams and cephalosporins exert bactericidal effects by inhibiting cell wall synthesis [38], while quinolones primarily act by inhibiting DNA gyrase and topoisomerase IV, thereby blocking DNA replication [39]. We hypothesize that mutations in CydDC impair normal heme transport, disrupting the electron transport chain (ETC)‐driven ATP production. This impairment may subsequently compromise cell wall and DNA synthesis, ultimately leading to altered resistance to drugs targeting these pathways (Figure 6). The identification of these key residues involved in heme transport provides crucial guidance for the precise design of antibacterial drugs. Alterations in heme biosynthesis can alter the activity of aminoglycoside antibiotics, such as gentamicin (GEN) [40, 41]. Furthermore, Qi et al. reported that *Lactococcus lactis* supplemented with heme developed moxifloxacin resistance more rapidly, possibly because heme addition induces the ETC, leading to reactive oxygen species (ROS) production [42]. This evidence underscores the potential role of heme in antibiotic resistance, further emphasizing the broad influence of its direct transporter, CydDC. Recent studies on inhibitors of heme or the electron transport chain have demonstrated the significant antibacterial potential of CydDC‐associated metabolic pathways [43, 44].

These findings also prompted us to consider a critical question: why do different mutants, all exhibiting impaired heme transport, exert distinct effects on different antibiotics? We hypothesize that CydDC may directly participate in drug efflux or possess multiple substrates. Among the antibiotics we tested, NIT, LEV, MEM, and CAZ share a similar backbone length with heme (Figure S11). The lateral cavity of CydDC could potentially accommodate these drug molecules directly and facilitate their efflux. Owing to the structural variations among these drug molecules, their interactions with CydDC also differ, leading to differential effects by specific mutants. Furthermore, the substrate‐binding cavity of CydDC might also accommodate other substrates, such as glutathione (GSH), in addition to heme. Although direct structural evidence for CydDC transporting substrates other than heme is currently lacking, numerous biochemical studies have demonstrated that CydDC is a crucial protein complex influencing bacterial redox homeostasis, potentially involved in transporting various important redox‐active molecules. Different mutants may not only affect heme transport but could also influence the transport of other substrates, thereby exerting distinct impacts on downstream physiological processes. However, this hypothesis requires further investigation.

Despite these findings, our study has certain limitations. The investigation of heme transport by CydDC is currently restricted to the initial binding states, and the detailed mechanisms of subsequent steps and the key sites involved remain to be elucidated. Furthermore, while this study focuses on the association between CydDC and resistance to commonly used antimicrobials, the functional scope of CydDC is likely broader, requiring more research evidence.

By integrating bioinformatics, structural biology, and biochemical experiments, this study systematically elucidates the key residues involved in heme transport by CydDC and further investigates the impact of impaired heme transport on bacterial antibiotic resistance. Our work provides new evidence for developing novel therapeutic strategies against drug‐resistant bacteria.

## 4 Experimental Section

### 4.1 Phylogenetic Analysis of cydC and cydD Orthologs

All orthologous genes of *cydC* and *cydD* were retrieved from the NCBI Gene database using NCBI‐Toolkit v24.1 (queries: esearch ‐db gene ‐query *cydC* and esearch ‐db gene ‐query *cydD*). Protein sequences with clearly annotated gene symbols corresponding to *cydC* or *cydD* were retained, and only one representative RefSeq entry was kept for sequences that were completely identical. In total, 906 *cydC* and 997 *cydD* sequences were obtained, representing 913 species across 47 taxonomic orders. Multiple sequence alignments were performed using MAFFT v7.526 (–maxiterate 1000 –localpair) [45], followed by automated alignment trimming with trimAl v1.5 under default settings [46]. Phylogenetic trees were constructed using IQ‐TREE v3 (‐nt 15, –seqtype AA) [47]. The best‐fit amino acid substitution model was determined according to the Bayesian information criterion, identifying LG+F+I+R10 as the optimal model. Visualization of the resulting phylogenetic trees was conducted using the R packages ggtree and ggtreeExtra [48, 49].

### 4.2 Conservation and Pocket Scoring Analysis for cydC and cydD Orthologs

To systematically evaluate the evolutionary conservation and functional relevance of the *cydD/C* complex, two complementary indices were calculated: the conservation score and the pocket score.

Conservation scores for *cydC* and *cydD* orthologs were computed using Python v3. For each aligned column *i* in the multiple sequence alignment, let *p_j_* denote the frequency of the *j*‐th amino acid in that column. Shannon entropy (*H_i_*) was calculated as:

$$H_{i}=-\sum_{j=1}^{20}p_{j}\ln\left(p_{j}\right)$$

where *H_i_* represents the information entropy of column *i*, using the natural logarithm. The entropy was then normalized as:

$$H_{i,\mathrm{norm}}=\frac{H_{i}}{\ln20}$$

and the conservation score (*C_i_*) was defined as:

$$C_{i}=1-H_{i,\mathrm{norm}}$$

The conservation score ranges from 0 to 1, where *C_i_* ≈ 1 indicates high conservation (dominance of a single amino acid), and *C_i_* ≈ 0 indicates high variability (lack of conservation). Columns with a non‐gap fraction below 0.6 or lacking any of the 20 standard amino acids were excluded from the calculation. The reference coordinate system was defined based on *Escherichia coli K‐12/MG1655*. The scores were visualized using ggplot2 package [50].

The pocket score was designed to quantify the overall conservation of the putative heme‐binding pocket based on the physicochemical properties of key residues. Specifically, each residue was assigned a score of 1 if the amino acid in each species retained a property consistent with the functional role inferred from reference species (*Escherichia coli K‐12/MG1655*), and 0 otherwise. For *CydC*, position 81 was scored as 1 when occupied by arginine or lysine (R or K), reflecting the preservation of positive charge; position 85 was assigned 1 for histidine (H); position 88 for threonine or serine (T or S); and position 132 for histidine (H). For *CydD*, position 247 received 1 if occupied by an aromatic residue such as phenylalanine, tyrosine, or tryptophan (F, Y, or W), and position 312 for histidine (H). The total pocket score for each species was calculated by summing these site‐specific scores. Higher scores indicate greater retention of biochemically compatible residues at key positions, reflecting stronger evolutionary conservation of the pocket, whereas lower scores suggest substitutions or alterations that may affect heme‐binding capacity.

### 4.3 Expression and Purification of CydDC

The gene fragments containing *cydD* and *cydC* from the *E. coli K‐12/MG1655* strain were amplified using PCR and then cloned into the pTrc99a plasmid. This resulted in the creation of the pTrc 99a‐CydDC construct, which included an octa‐histidine (8×His) tag at the C terminus of CydC. pTrc 99a‐CydDC_His_ was used as the template to carry out site‐directed mutation PCR reaction and introduce CydDC mutation. The modified plasmid was introduced into *E. coli* C43 (DE3) cells for protein expression.

The transformed bacterial cells were cultivated at 37°C in LB medium supplemented with 100 µg mL^−1^ ampicillin. Protein production was initiated by adding 0.2 mm Isopropyl β‐D‐thiogalactoside (IPTG) at 20°C for 12 h, once the culture's optical density at 600 nm (OD_600_) reached 0.8. Subsequently, cell pellets were harvested via centrifugation (4000 rpm, 15 min), resuspended in lysis bufferA (20 mm hepes, pH 7.8, 300 mm NaCl, and 10% glycerol) containing 0.1 mm phenylmethylsulfonyl fluoride (PMSF). The cells were lysed and the cell membranes were isolated through ultracentrifugation at 140 000 *g* for 1 h at 4°C. The membranes were then solubilized using bufferA containing 1% n‐dodecyl‐β‐D‐maltopyranoside (DDM, Lablead) for 1 h and centrifugation. The suspension containing solubilized protein was purified using a HisTrap HP column (5 mL, GE HealthCare). The bound protein was eluted with bufferA containing 300 mm imidazole and 0.02% lauryl‐maltose‐neopentyl‐glycol (LMNG, Anatrace). Further purification was performed on a Superdex 200 Increase 10/300 column (GE Healthcare) using bufferB (20 mm hepes, pH 7.8, 150 mm NaCl) supplemented with 0.02% LMNG. The presence of each protein in mutants was detected by western blot using mouse anti‐Myc (1:1000 dilution; A5963, Sigma) and anti‐Flag monoclonal antibody (1:5000 dilution; F3165, Sigma).

### 4.4 Nanodiscs Reconstitution

The reconstitution process of purified CydDC into nanodiscs was adapted from established protocols [51]. In brief, *E. coli* polar lipid extracts (Avanti Polar Lipids) in chloroform was dried under argon gas and vacuum‐stored overnight. The resulting lipid film was re‐suspended in nanodisc bufferB with 25 mm sodium cholate, sonicated in a water bath until homogeneous. CydDC, the membrane scaffold protein MSP1D1, and *E. coli* polar lipids were mixed at a molar ratio of 1:2:100 in nanodisc buffer and incubate at 4°C for 1 h. Detergent removal was achieved by incubating with 0.6 g mL^−1^ Bio‐Beads SM‐2 (Bio‐Rad) at 4°C for 2 h. The nanodisc‐reconstituted CydDC underwent further purification via size exclusion chromatography using a Superdex 200 column. The purity of CydDC within the nanodisc was evaluated using sodium dodecyl sulfate‐polyacrylamide gel electrophoresis (SDS‐PAGE) and negative stain electron microscopy.

### 4.5 ATP Hydrolysis Assays

The ATPase activity of CydDC was assessed utilizing the ATPase Activity Assay Kit (Bioassay Systems) as per the manufacturer's guidelines. In brief, 1 µg of CydDC was exposed to a mixture of 25 mm Tris, pH 7.8, 150 mm NaCl, 4 mm ATP, and 4 mm MgCl_2_ for 30 min at 37°C. The reaction was halted by introducing 200 µl of the reagent (ATPase Assay Kit) into each sample, followed by an additional 30‐minute incubation period. Subsequently, the absorbance at 600 nm was recorded. A standard curve for phosphate was established using the stock solutions provided in the kit to determine the total concentration of released phosphate. The ATPase activities of all samples were determined by calculating the mean value based on the linear regression of the standards.

### 4.6 Cryo‐EM Sample Preparation and Data Acquisition

To obtain CydDC‐nanodisc complexes bound with different ligands, the purified nanodisc‐reconstituted protein after size exclusion chromatography was first concentrated to 4 mg mL^−1^ and centrifuged at 15 000 rpm for 6 min at 4°C. For ATP‐ or AMP‐PNP‐bound CydDC samples, ligands were added to a final concentration of 2 mm along with 5 mm MgCl_2_. The mixture was gently homogenized, incubated at room temperature for 20 min, and then centrifuged at 15 000 rpm for 30 min at 4°C. The protein supernatant was subsequently transferred to a new microcentrifuge tube. For heme‐bound CydDC samples, heme was supplemented to the pre‐concentrated CydDC‐nanodisc sample at a final concentration of 50 µm. After gentle homogenization and incubation at room temperature for 20 min, the mixture was centrifuged at 15 000 rpm for 30 min at 4°C, and the resulting protein solution was transferred to a new microcentrifuge tube for downstream experiments.

The purified protein (3 µL) or mixture with ligands was applied onto glow‐discharged (60 s) Quantifoil grids (Au, 1.2/1.3 µm size/hole space, 300 mesh). Subsequently, the grids were blotted for 2 s at 100% humidity at 4°C, and then immersed in liquid ethane cooled by liquid nitrogen using a Vitrobot Mark IV (Thermo Fisher). Cryo‐EM data were collected using a Titan Krios microscope (Thermo Fisher) equipped with a Quantum GIF energy filter featuring an energy slit of 20 eV (Gatan), operating at 300 kV. A Gatan K2 Summit direct electron detector was employed to record movies in counting mode on the Krios at a nominal magnification of 165 000× with a pixel size of 0.85 Å. Defocus values ranged from ‐1.7 to ‐1.2 µm. Each stack underwent a 6‐second exposure and was dose‐fractionated into 30 frames, with a total dose of 59.7 e^−^ Å^−2^. Movie stacks were automatically collected using EPU software.

### 4.7 Cryo‐EM Data Processing

The image processing of apo‐CydDC is depicted in Figures S3 and S4. In summary, data processing was primarily conducted using RELION (version 3.1) [52]. Initially, for apo of CydDC, 3483 dose‐fractionated images underwent motion correction with MotionCor2 [53], followed by CTF estimation utilizing CTFFIND4 [54]. Subsequently, 3,437 micrographs were processed with EMAN2.31 for neural network particle picking [55], resulting in 534,633 particles. Rounds of reference‐free 2D classification were executed in Relion (version 3.1). From there, 101,174 particles underwent ab‐initio reconstruction and heterogeneous refinement involving 6 classes, unveiling clear secondary structure in the transmembrane region within one of the classes. For further refinement, 49 171 particles were selected for homogeneous refinement, non‐uniform refinement, and local refinement. The EM density map was sharpened using a negative B‐factor, automatically determined in cryoSPARC [56], aided by a Guinier plot. Reconstructions’ resolution was evaluated using the Fourier shell correlation (FSC) criterion with a threshold of 0.143 in cryoSPARC. Lastly, the local resolution of the final map was estimated in cryoSPARC.

### 4.8 Model Building and Refinement

The Alphafold2‐predicted model served as an initial reference. The model was docked into the electron density using UCSF Chimera [57]. Then, each individual residue was manually examined and adjusted to fit the map in Coot [58]. Subsequently, the model was refined against the corresponding map in PHENIX [59]. MolProbity was used to evaluate the stereochemistry and geometry of the structure. Figures were prepared in USCSF Chimera and UCSF ChimeraX.

### 4.9 Analysis of CydC and CydD Expression between Drug‐Resistant and Sensitive Bacteria

The drug resistance‐related dataset of *Escherichia coli* (*E. coli*) was downloaded from the NCBI Gene Expression Omnibus (GEO) database, with the dataset ID number GSE96706. In this dataset, we selected data‐related to 7 antibiotics with different antibacterial mechanisms, including ampicillin (AMP), chloramphenicol (CHL), cefprozil (CPR), doxycycline (DOX), erythromycin (ERY), cefoxitin (FOX), kanamycin (KAN), nalidixic acid (NAL), nitrofurantoin (NIT), tetracycline (TET), tobramycin (TOB) and trimethoprim (TRM). Sensitive strains were used for control. Raw data of the above groups was downloaded and underwent steps such as filtering, alignment, quantification and so on. The DESeq2 package (version 1.38.3) was applied for differential analysis [60]. Genes with adjusted *p* values (*p*_adj_) ≤ 0.05 were considered differentially expressed genes. Bean plots were used to visualize the data.

### 4.10 Susceptibility Testing of CydDC Mutants

To delve deeper into the relationship between CydDC and antibiotic resistance, we opted for antibiotics frequently utilized in clinical settings to combat Gram‐negative bacteria for our inhibition zone experiments. We used the λ‐Red technology to knock out the *cydDC* gene of the *E. coli MG1655* strain to obtain the *MG1655(ΔCydDC)* strain. This strain was electrocompetent and then electroporated into the pTrc 99a‐CydDC and mutant plasmid and coated on LB agar plates with 50 µg mL^−1^ ampicillin for 24 h. Positive clones were picked, incubated overnight with LB liquid medium at 37°C, then adjust to a 0.5 McFarland turbidity using physiological saline. The bacterial solution was evenly coated on Mueller‐Hinton Broth (MHB) agar plates, pasted with antimicrobial susceptibility tablets, and incubated at 37°C for 18–24 h, and the size data of the inhibition zone were collected.

### 4.11 Affinity Test by Biolayer Interferometry

Biotinylated CydDC wildtype and mutants were diluted to a concentration of 1 mg mL^−1^ in bufferB supplemented with 0.02% LMNG and then applied to super streptavidin biosensors, resulting in a total shift of 1 nm using OCTET K2 (ForteBio) [61]. The loaded tips were immersed in serial dilutions of heme (ranging from 25 to 1.56 µm) for 180 s, followed by dissociation in the modified kinetics buffer for 300 s. The data was analyzed using Data Analysis HT (version 11.1.2.48), and the binding curves were fitted and visualized using Graphpad Prism (version 10.1.2). The area under the curves was calculated by summing the binding response values for each data point, with a binding detection limit of 0.01 nm.

## Author Contributions

H.D. and X.T. conceived and designed the experiments. L.Y. and C.Z. designed and constructed the CydDC complex vector for protein expression. L.L., G.H., and M.L. performed the bioinformatic analysis of CydDC. L.Y., C.Z., and J.Z. purified the CydDC complex and prepared the final samples for data collection and affinity assays. Y.L. screened the samples for data collection. Y.L. collected and processed cryo‐EM data of CydDC complex. H.D. and Y.L. built, refined and validated atomic coordinate model of CydDC complex. H.D., X.T., G.H., L.Y., M.L., C.Z., J.Z., and Y.C. participated in data and manuscript preparation. All authors discussed the results and commented on the manuscript.

## Conflicts of Interest

The authors declare no conflicts of interest.

## Supporting information




**Supporting File**: advs76081‐sup‐0001‐SuppMat.docx.

**Supporting Table**: advs76081‐sup‐0002‐TableS1.xlsx.

## Data Availability

The high‐throughput sequencing data used in this work are public and their dataset IDs are provided in the Materials and methods section. Electron microscopy density maps and atomic models have been deposited in the Electron Microscopy Data Bank (EMDB) and PDB, respectively, with accession codes EMD‐55775 and 9TBY; EMD‐67183 and 9XSM; EMD‐67273 and 9XUO; EMD‐67055 and 9XNP; EMD‐67054 and 9XNO.
