# Supplementary material for: Conformational Snapshots of CydDC in a Native Lipid Bilayer Coupling Heme Transport to Antibiotic Resistance
Source: Adv Sci (Weinh). 2026 Jun 11:e76081. Online ahead of print. doi: 10.1002/advs.76081 (PMC13337084; doi:10.1002/advs.76081)
Supplement: Supplementary file 1 — Supporting File: advs76081‐sup‐0001‐SuppMat.docx. [file ADVS-9999-e76081-s001.docx]

**Supporting Information for**

**Conformational snapshots of CydDC in a native lipid bilayer coupling heme transport to antibiotic resistance**

*Lili Yang, Changbin Zhang, Mengyuan Lyu, Yongbo Luo, Jierou Zhang, Yujiao Chen, Lintao Luo, Wen Qiao, Xiangyangpeng Li, Yu Zhou, Zhongling Wei, Yuling Xiao, Qian Niu, Juan Zhou, Guanglin He*, Binwu Ying*, Zhaoming Su*, Hao Chen*, Xiaodi Tang*, Haohao Dong**

The file includes:

Figures S1-S11

Table S2-S3


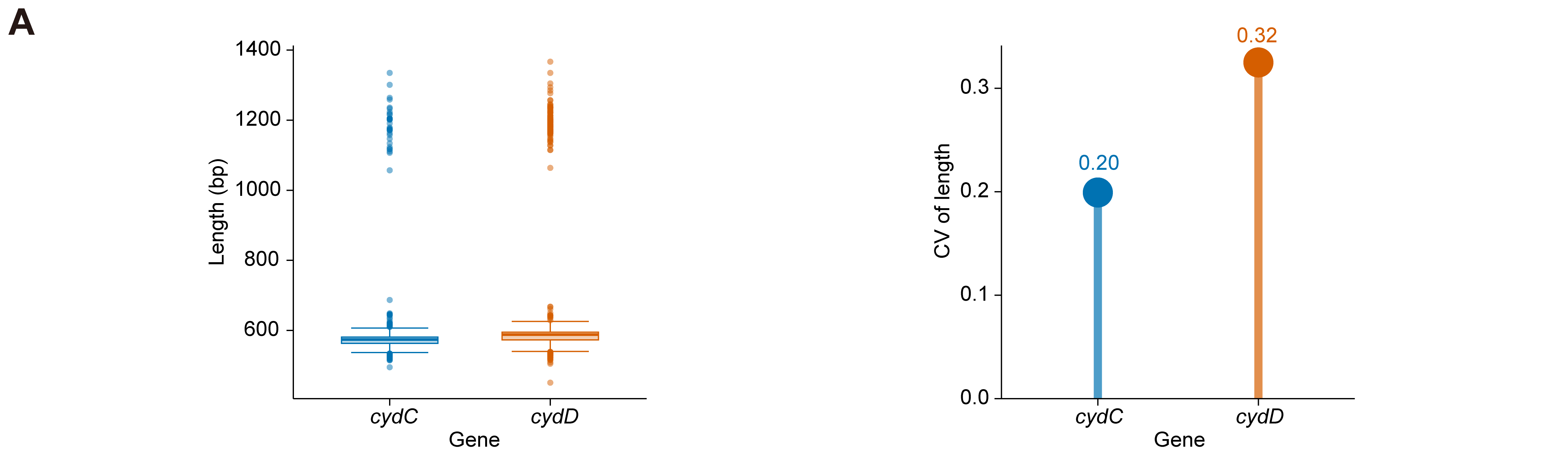


**Figure S1. Length distribution and variation of *cydC* and *cydD* genes across species**

Left: boxplot showing the distribution of gene lengths (bp) for *cydC* and *cydD*. Each box represents the interquartile range, with the horizontal line indicating the median value; dots denote outliers. Right: coefficient of variation of gene length for *cydC* and *cydD*.


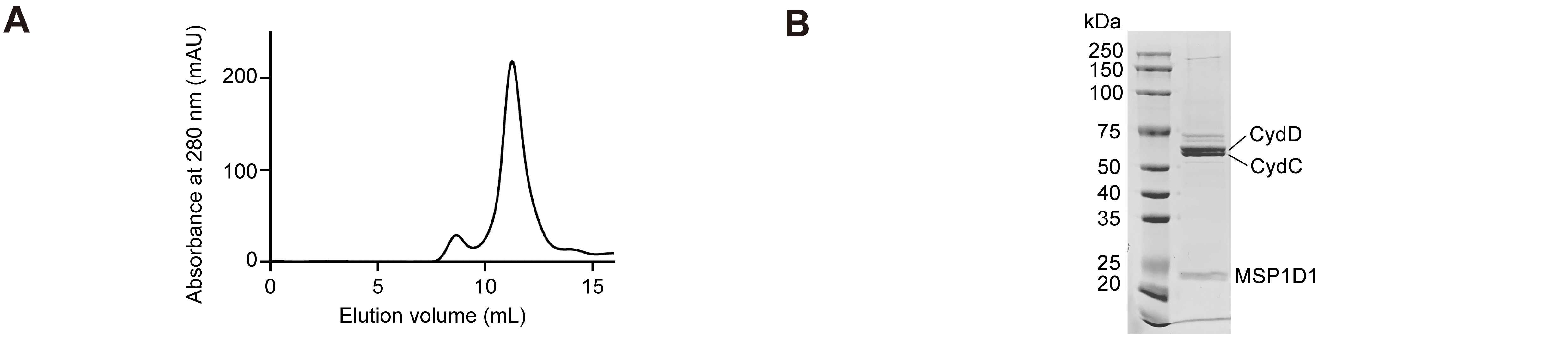


**Figure S2.** **Purification results of CydDC and ATPase activity assay.**

**A.** Size exclusion chromatography results of CydDC.

**B.** Coomassie blue stained SDS- PAGE of CydDC within nanodisc.


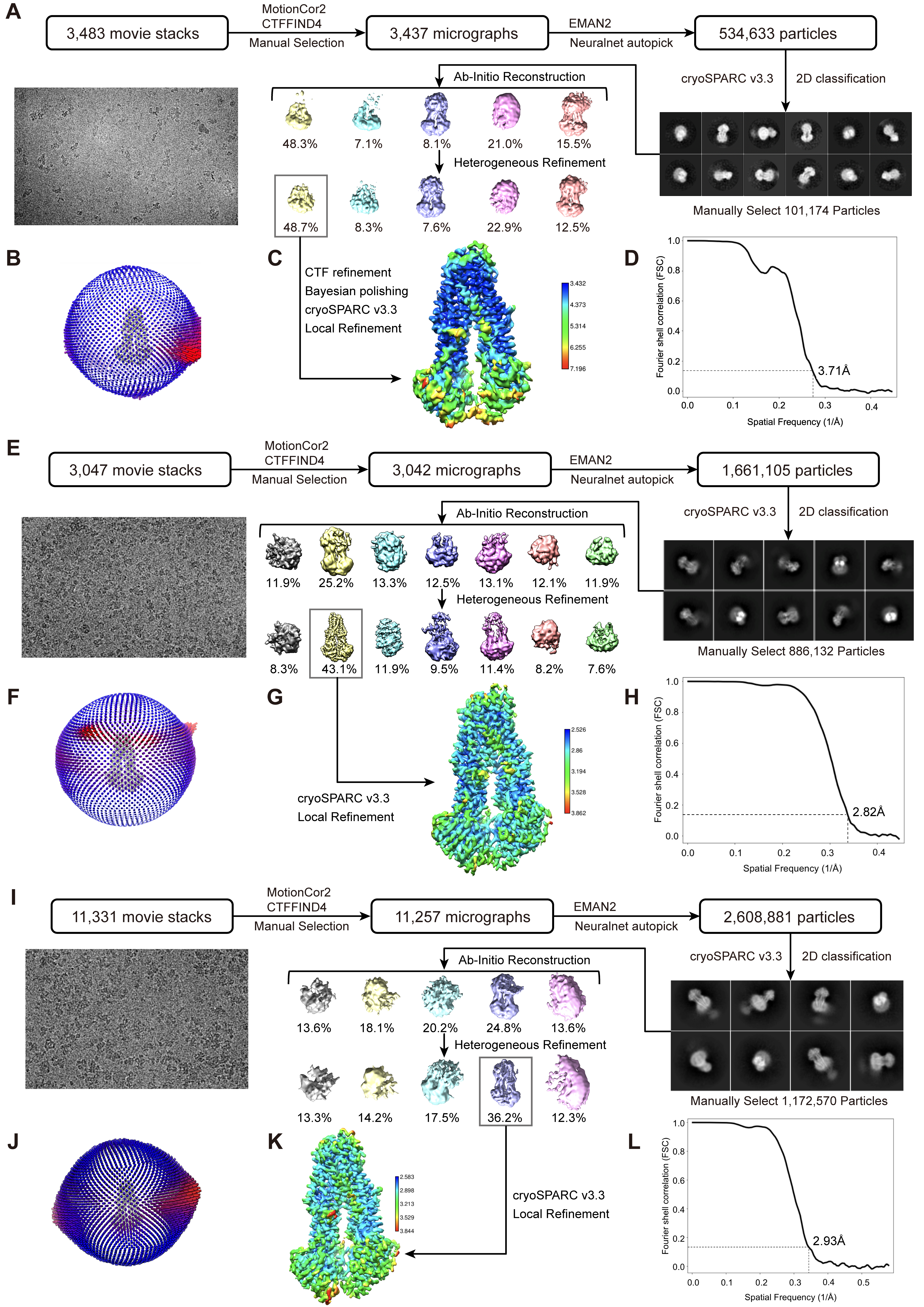


**Figure S3. Cryo-EM processing flow chart of** **apo, ATP-bound, AMP-PNP-bound CydDC.**

**A, E, I.** Micrograph of apo-CydDC single particles after drift correction and dose-weighting, followed by 2D classifications, 3D classification, selections and 3D refinement of apo, ATP-bound, AMP-PNP-bound CydDC.

**B, F, J.** Angular distribution of the cryo-EM particles included in the final 3D reconstruction of apo, ATP-bound, AMP-PNP-bound CydDC.

**C-D, G-H, K-L.** Cryo-EM map colored according to local resolutions and FSC curve of apo, ATP-bound, AMP-PNP-bound CydDC.


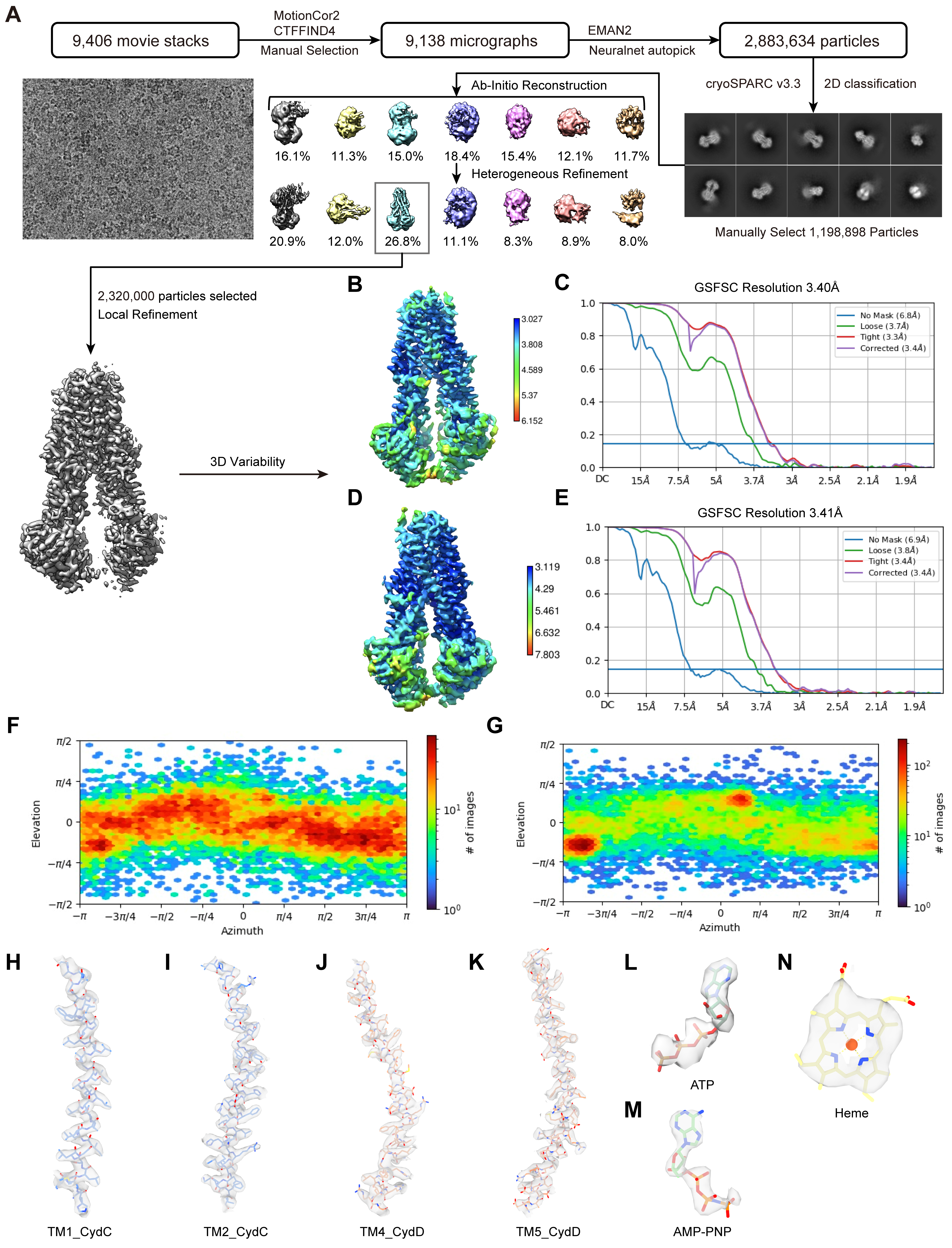


**Figure S4. Cryo-EM processing flow chart of heme-bound CydDC.**

**A.** Micrograph of heme-bound CydDC single particles after drift correction and dose-weighting, followed by 2D classifications, 3D classification, selections and 3D refinement.

**B-G.** Presentation of the density map (B, D), gold-standard Fourier shell correlation (FSC) curve (C, E) and Euler angle distribution (F, G) for the classified particles of heme-bound CydDC.

**H-N.** Cryo-EM maps of the selected CydDC and heme, ATP, AMP-PNP.


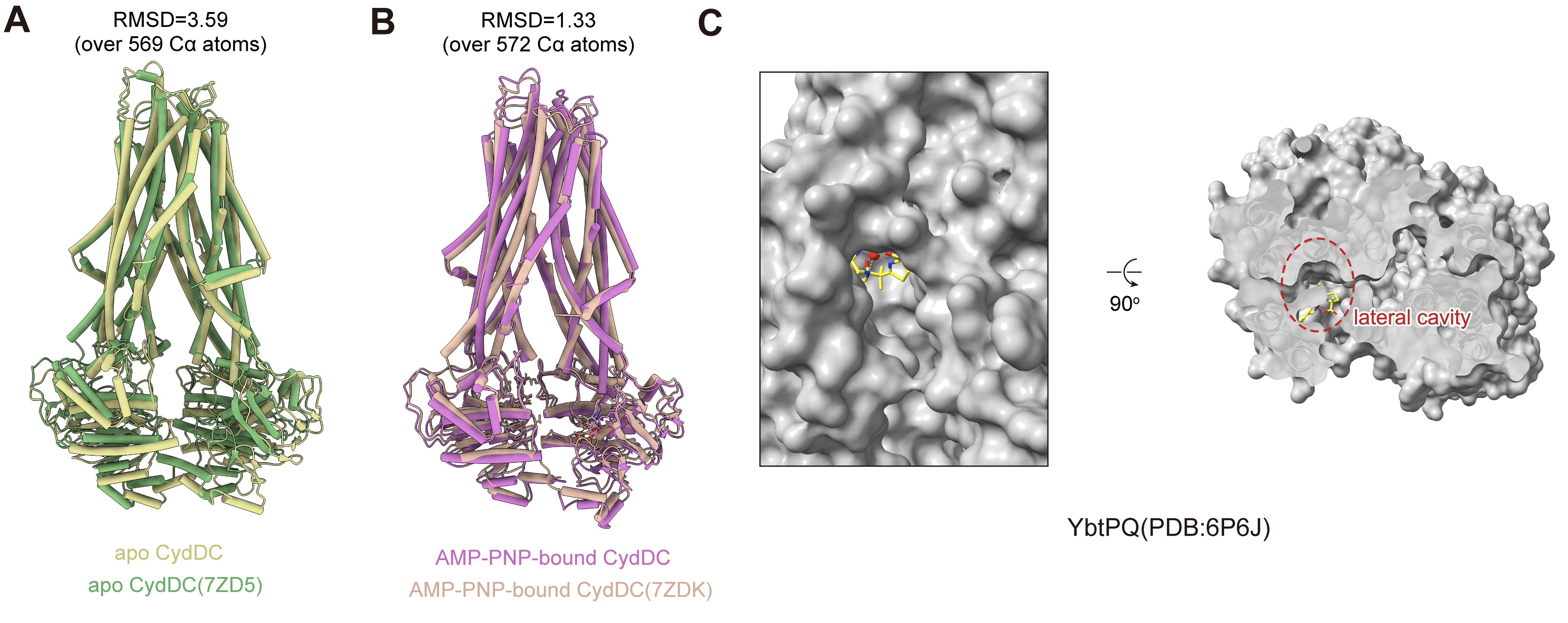


**Figure S5. Superimposition of CydDC and Heme binds into the lateral cavity of CydDC.**

**A-B.** Superimposition of CydDC in this study and reported CydDC.

**C.** Lateral cavity of YbtPQ


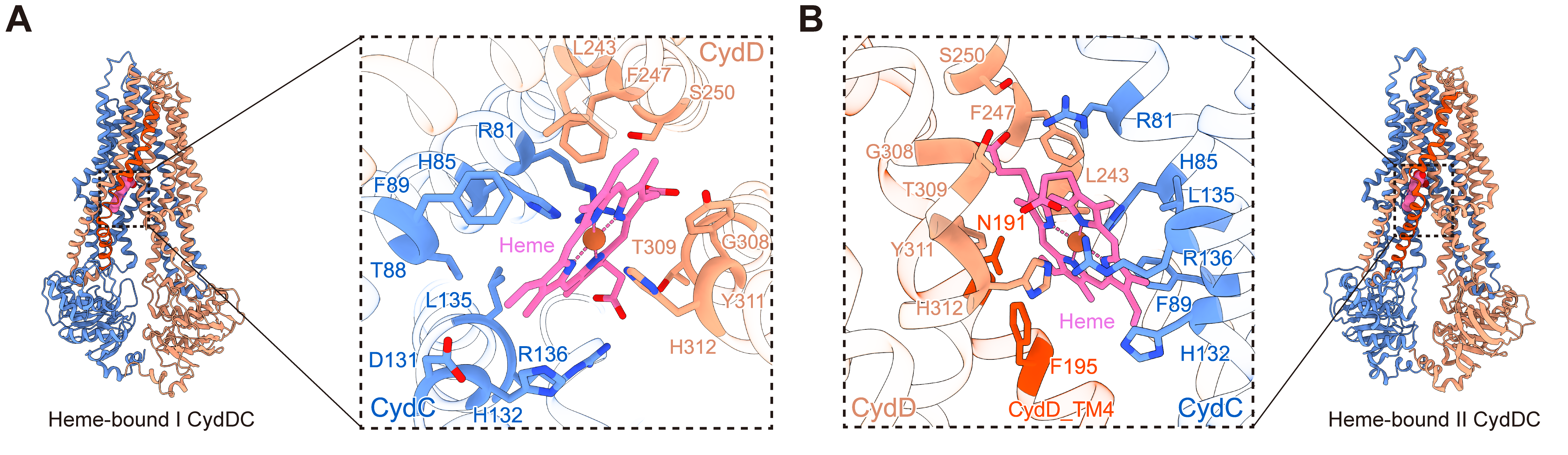


**Figure S6. Details of heme bingding to CydDC.**

**A-B.** All of the residues interacting with heme in heme-bound I (A) and heme-bound II CydDC (B).


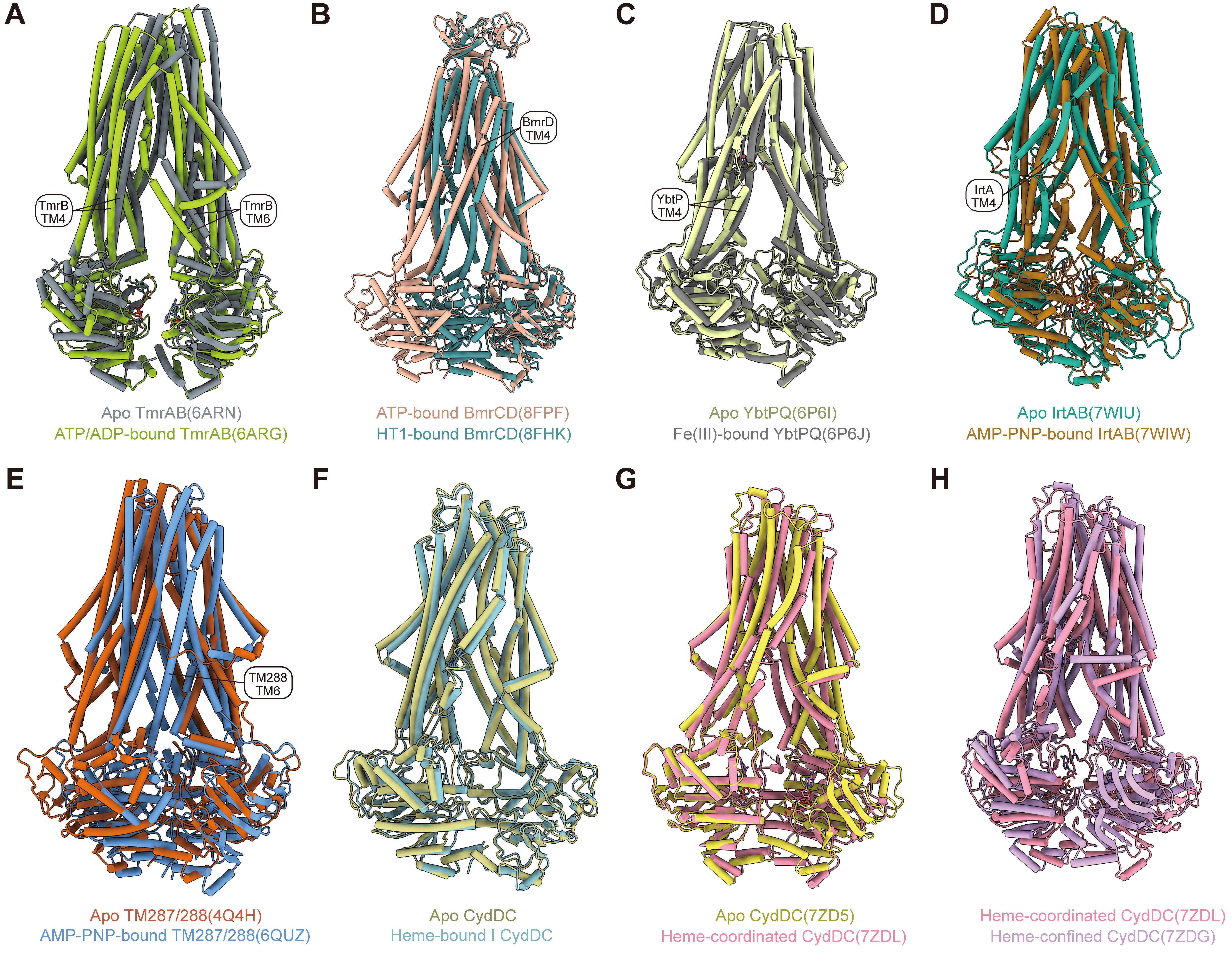


**Figure S7. Superimpositions of heterdimeric type IV ABC transpoters.**

**A-E.** Superimpositions of TmrAB (A), BmrCD (B), YbtPQ (C), IrtAB (D), TM287/288 E) between apo with nucleotid or substrate binding.

**F-H.** Superimpositions of CydDC in different states.


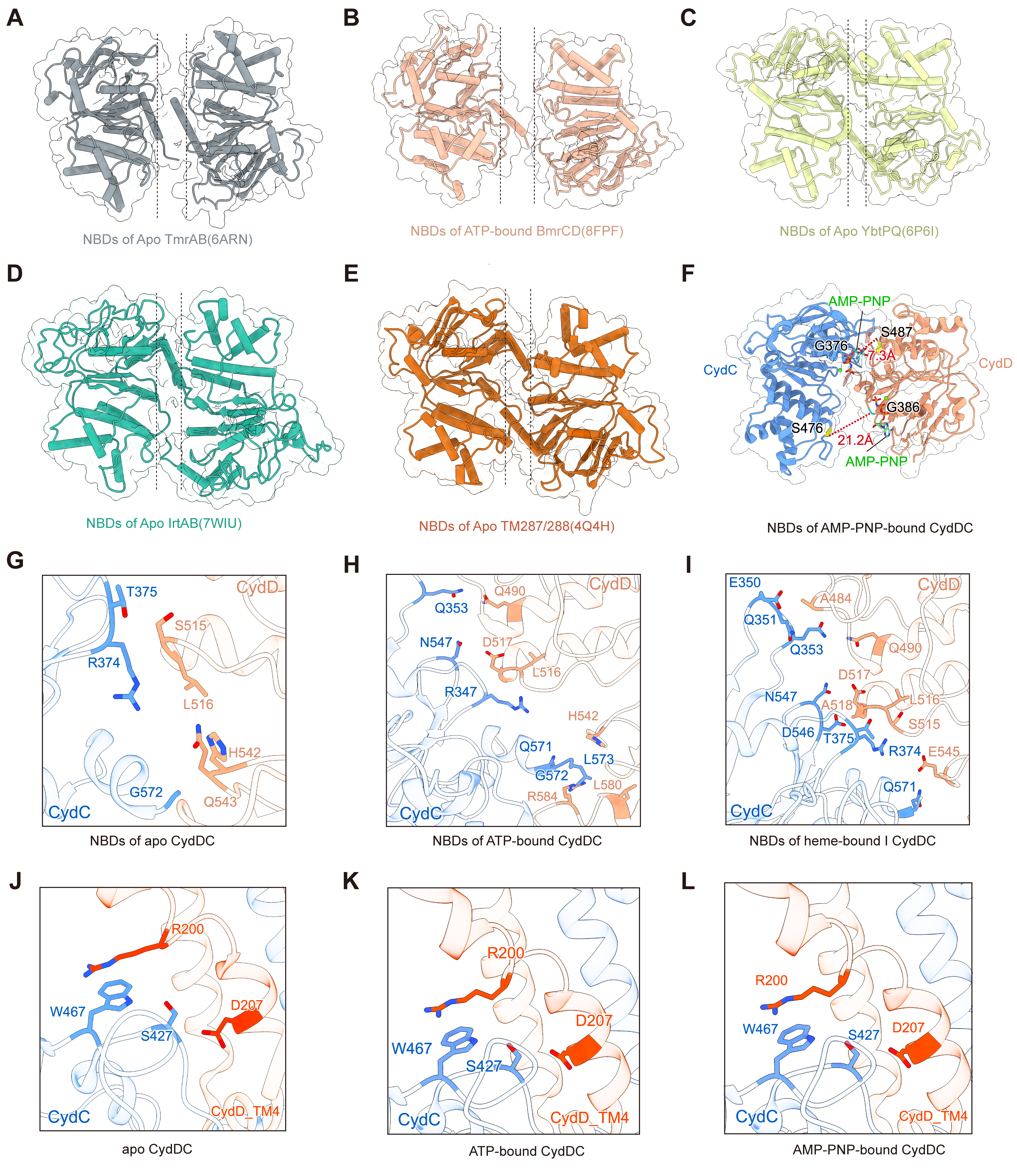


**Figure S8. Analysis of NBDs interactions of CydDC and other** **heterdimeric type IV ABC transpoters.**

**A-F.** Distances analysis of heterdimeric type IV ABC transpoters in ATP-bound or apo state.

**G-I.** Interactions between NBDs of CydDC.

**J-L.** Interactions between NBD of CydC and TM4 of CydD.


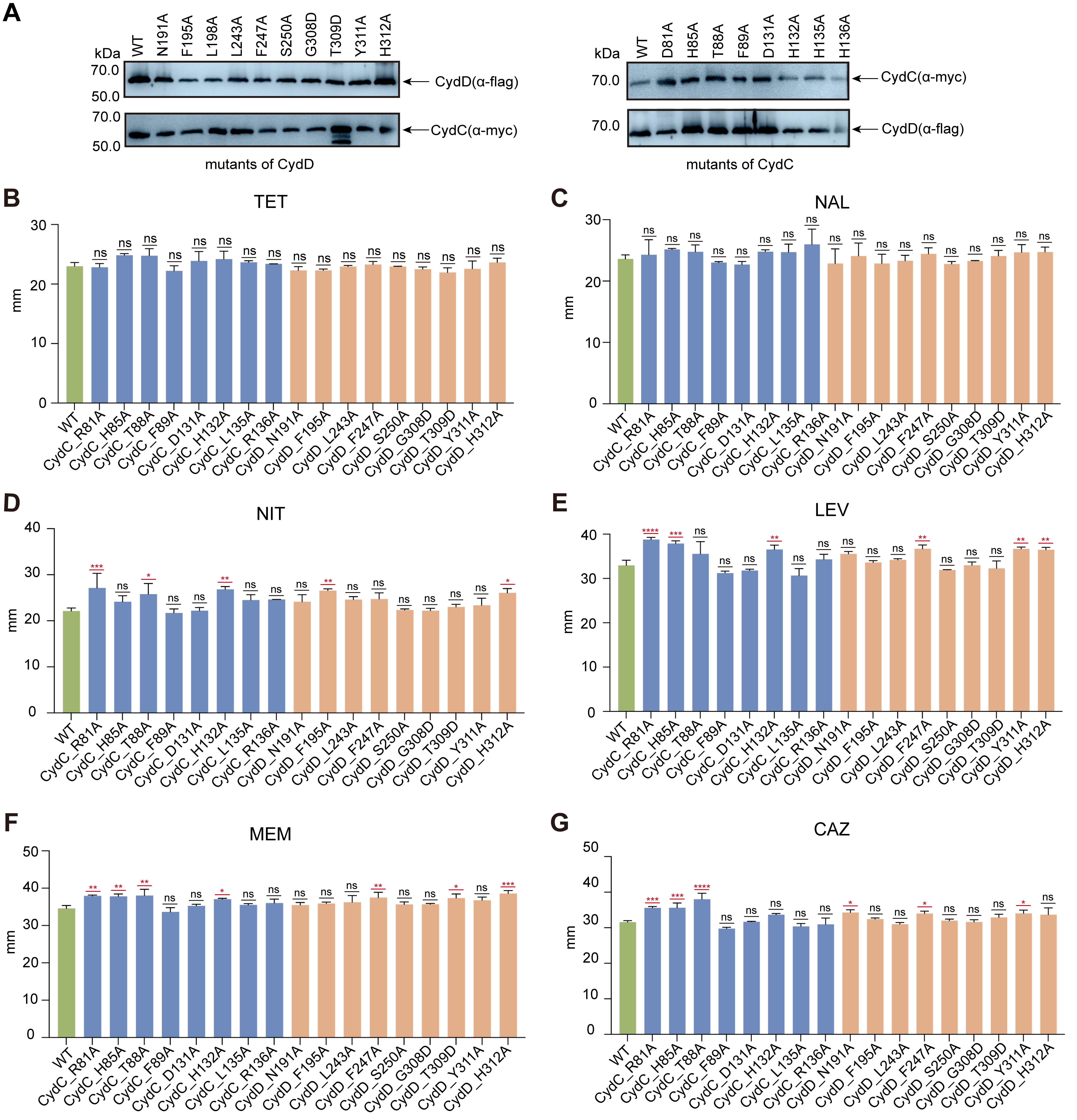


**Figure S9. Susceptibility testing of CydDC mutants.**

**A.** Western blot analysis of CydDC mutants

**B-G.** Zone of clearance about CydDC mutant with different antibiotics. Adjusted *p* values (*p_adj_*) are marked with red asterisks for significant differences. ns: no significant differences. WT: wildtype. WT CydDC is colored with green; Mutants with mutations in CydC and CydD are colored with cornflower blue and light salmon, respectively. **p_adj_*< 0.05, ***p_adj_*< 0.01, ****p_adj_*< 0.001, *****p_adj_*< 0.0001.


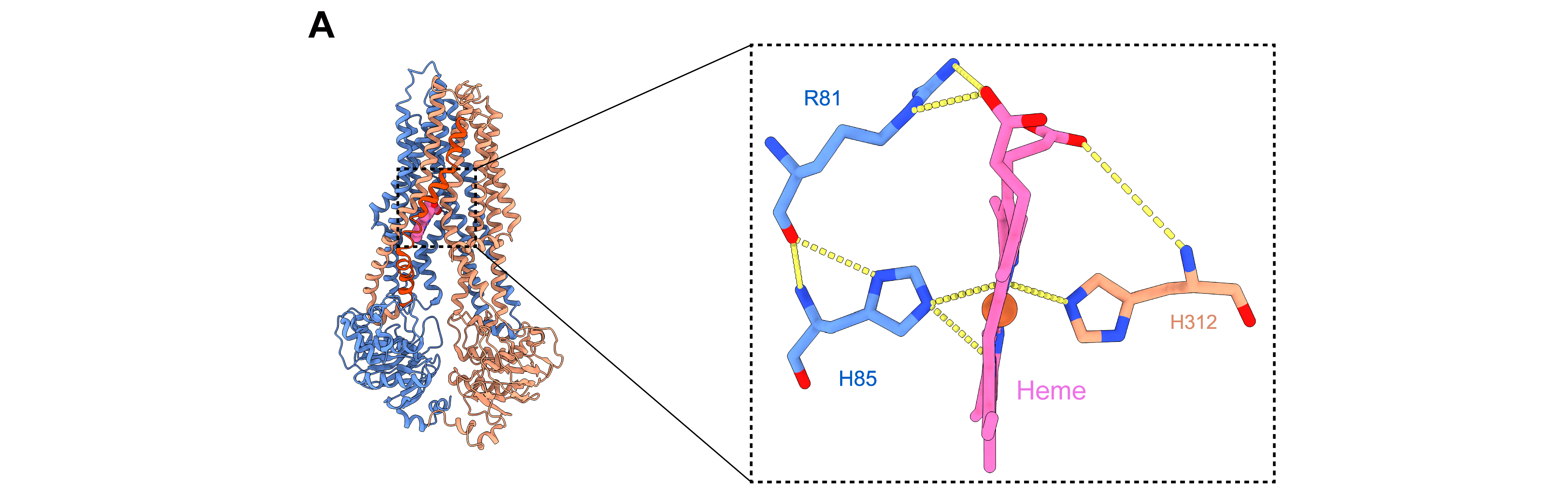


**Figure S10. Key negatively charged residues of heme binding to CydDC.**

**A.** Hydrogen bonds between heme and CydC^R81^, CydC^H85^, CydD^H312^. Hydrogen bonds are shown as yellow dashed lines.

**
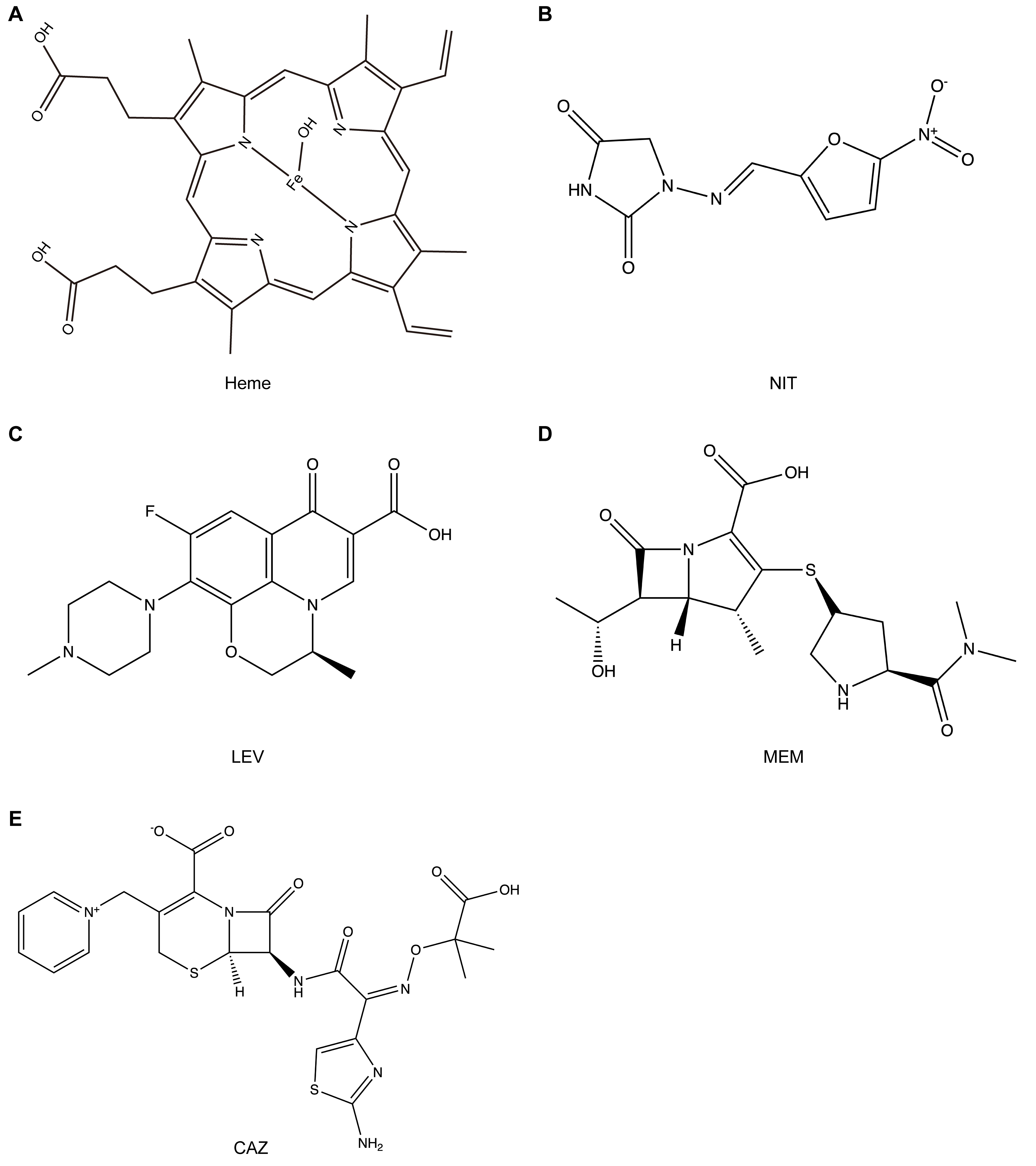
**

**Figure S11.** **Molecular structure of Heme and tested antibiotics.**

**A-E.** Molecular structure of Heme (A), nitrofurantoin (NIT) (B), levofloxacin (LEV) (C), meropenem (MEM) (D), ceftazidime (CAZ) (E).

**Table S1 | Taxonomic information of the 913 representative prokaryotic species included in this study.**

This table is provided as a separate file.

**Table S2 | Summary statistics of gene length and conservation metrics for cydC and cydD**

| **Gene** | cydC | cydD |
| --- | --- | --- |
| **No. of species (n)** | 906 | 997 |
| **Median gene length (bp)** | 574 | 588 |
| **Coefficient of variation (CV)** | 0.199 | 0.325 |
| **Mean pocket score** | 547.18 | 555.12 |
| **SD (pocket score)** | 21.54 | 24.64 |
| **Mean completeness** | 0.955 | 0.944 |
| **SD (completeness)** | 0.038 | 0.042 |

**Table S3 | Cryo-EM data collection, refinement and validation statistics**

|  | Apo CydDC (EMD-55775,  PDB 9TBY) | AMP-PNP-bound CydDC  (EMD-67054,  PDB 9XNO) | ATP-bound CydDC (EMD-67055, PDB 9XNP) | Heme-bound I CydDC (EMD-67183,  PDB 9XSM) | Heme-bound II CydDC (EMD-67273,  PDB 9XUO) |
| --- | --- | --- | --- | --- | --- |
| **Data collection and**  **processing** | | | | | |
| Magnification | 130,000× | 165,000× | 130,000× | 165,000× | |
| Voltage (kV) | 300 | 300 | 300 | 300 | |
| Electron exposure (e–/Å^2^) | 1.70 | 2.23 | 1.80 | 2.23 | |
| Defocus range (μm) | 0.9~2.7 | 0.5~2.6 | 1.3~2.4 | 0.5~2.6 | |
| Pixel size (Å) | 1.1 | 0.85 | 0.85 | 0.85 | |
| Symmetry imposed | C1 | C1 | C1 | C1 | |
| Initial particle images (no.) | 534,633 | 2,608,881 | 1,661,105 | 2,883,634 | |
| Final particle images (no.) | 49,171 | 424,631 | 434,665 | 23,007 | 24,909 |
| Map resolution (Å)  FSC threshold | 3.71  0.143 | 2.93  0.143 | 2.82  0.143 | 3.40  0.143 | 3.41  0.143 |
| Map resolution range (Å) | 3.4~7.1 | 2.5~3.8 | 2.5~3.8 | 3.0~6.1 | 3.1~7.8 |
|  | | | |  |  |
| **Refinement** | | | | | |
| Initial model used | - | - | - | - | - |
| Initial model CC  Model resolution (Å)  FSC threshold | 0.77  4.0  0.5 | 0.84  3.1  0.5 | 0.81  3.0  0.5 | 0.84  3.6  0.5 | 0.73  3.8  0.5 |
| Map sharpening *B* factor (Å^2^) | -86.2 | -106.5 | -102.6 | -72.0 | -74.1 |
| Model Composition | | | |  |  |
| Non-hydrogen atoms  Protein residues  Ligands | 8909  1149  0 | 8998  1154  2 | 9047  1156  2 | 8977  1154  1 | 9041  1160  1 |
| *B* factor (Å^2^) | | | |  |  |
| Protein  Ligand | 120.35 | 44.64  53.92 | 60.71  65.47 | 102.60  86.89 | 95.34  60.36 |
| R. m. s. devations | | | |  |  |
| Bond lengths (Å)  Bond angles (°) | 0.002  0.508 | 0.008  0.776 | 0.005  0.674 | 0.003  0.646 | 0.002  0.501 |
| **Validation** | | | |  |  |
| Refined model CC  MolProbity score  Clashscore  Poor rotamers (%) | 0.76  1.65  4.33  0.00 | 0.87  1.64  4.09  0.00 | 0.83  1.63  3.46  0.00 | 0.86  1.91  7.35  0.00 | 0.75  1.71  5.10  0.00 |
| Ramachandran plot | | | |  |  |
| Favored (%)  Allowed (%)  Disallowed (%) | 93.28  6.72  0.00 | 93.04  6.87  0.00 | 91.84  8.16  0.00 | 91.48  8.52  0.00 | 93.17  6.83  0.00 |
